# Pharmaceutical integrated stress response enhancement protects oligodendrocytes and provides a potential multiple sclerosis therapeutic

**DOI:** 10.1038/ncomms7532

**Published:** 2015-03-13

**Authors:** Sharon W. Way, Joseph R. Podojil, Benjamin L. Clayton, Anita Zaremba, Tassie L. Collins, Rejani B. Kunjamma, Andrew P. Robinson, Pedro Brugarolas, Robert H. Miller, Stephen D. Miller, Brian Popko

**Affiliations:** 1 Department of Neurology, The University of Chicago Center for Peripheral Neuropathy, The University of Chicago, Chicago, Illinois 60637, USA; 2 Department of Microbiology-Immunology and Interdepartmental Immunobiology Center, Northwestern University Feinberg School of Medicine, Chicago, Illinois 60611, USA; 3 Department of Neurosciences, Case Western Reserve University School of Medicine, Cleveland, Ohio 44106, USA; 4 Myelin Repair Foundation, Saratoga, California 95070, USA

## Abstract

Oligodendrocyte death contributes to the pathogenesis of the inflammatory demyelinating disease multiple sclerosis (MS). Nevertheless, current MS therapies are mainly immunomodulatory and have demonstrated limited ability to inhibit MS progression. Protection of oligodendrocytes is therefore a desirable strategy for alleviating disease. Here we demonstrate that enhancement of the integrated stress response using the FDA-approved drug guanabenz increases oligodendrocyte survival in culture and prevents hypomyelination in cerebellar explants in the presence of interferon-γ, a pro-inflammatory cytokine implicated in MS pathogenesis. *In vivo*, guanabenz treatment protects against oligodendrocyte loss caused by CNS-specific expression of interferon-γ. In a mouse model of MS, experimental autoimmune encephalomyelitis, guanabenz alleviates clinical symptoms, which correlates with increased oligodendrocyte survival and diminished CNS CD4+ T cell accumulation. Moreover, guanabenz ameliorates relapse in relapsing-remitting experimental autoimmune encephalomyelitis. Our results provide support for a MS therapy that enhances the integrated stress response to protect oligodendrocytes against the inflammatory CNS environment.

## Abstract (web summary)

Current multiple sclerosis treatments focus on prevention of immune attack on oligodendrocytes and myelin. Here the authors show a different strategy to ameliorate disease in several mouse models, protecting oligodendrocytes from inflammation-induced death with an FDA-approved drug, guanabenz.

## Graphical abstract

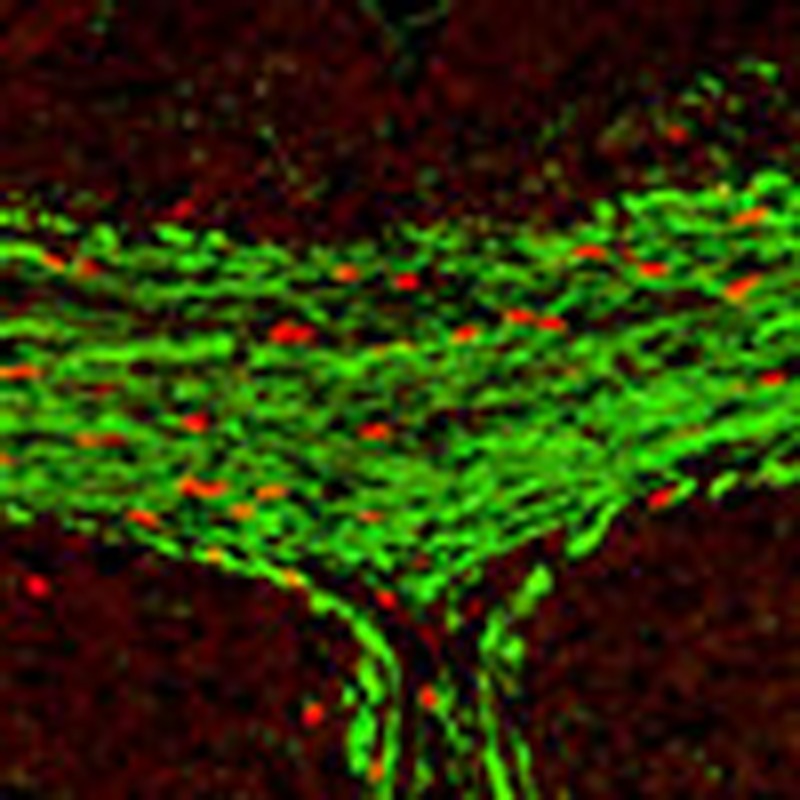

Multiple sclerosis (MS) is a chronic inflammatory disease of the central nervous system (CNS) in which initial relapsing-remitting neurological symptoms progress to more severe functional loss in the majority of patients^1^. While the aetiology of MS is yet unknown, its pathological hallmarks include immune-mediated destruction of axon-supporting myelin and oligodendrocytes, the cells that produce and maintain myelin^2^. This loss of trophic support from oligodendrocytes and myelin leaves axons functionally compromised and unprotected, and recent studies have demonstrated that progressive axonal loss significantly contributes to irreversible neurological disability in MS patients^3^. Although current MS therapies have alleviated the relapsing phases of disease with varying degrees of success, their solely immunomodulatory focus has demonstrated limited ability to inhibit the progression of MS disability^4^. The focus of the field has therefore shifted to finding reparative approaches that might be used in combination with anti-inflammatory treatments to restore axonal support and function^5^. Recent efforts to enhance oligodendrocyte differentiation^6^ and remyelination^7^,^8^ capabilities, for example, have demonstrated exciting potential in further alleviating disease.

In addition to dampening inflammation and enhancing repair, however, a largely overlooked protective approach—inhibiting the initial loss of oligodendrocytes and myelin—may provide the remaining complementary factor needed to inhibit MS progression. While it is well accepted that healthy oligodendrocytes are required for axonal support, function and long-term survival^9^,^10^, the exact timing and nature of oligodendrocyte loss during MS disease progression remains poorly understood. Nevertheless, studies in experimental autoimmune encephalomyelitis (EAE), a mouse model of MS, suggest that the extent of oligodendrocyte loss correlates with the degree of myelin and axonal loss^11^. Meanwhile, two independent methods of inhibiting apoptosis in oligodendrocytes in EAE both resulted in significantly reduced demyelination, ameliorated disease severity^12^ and limited axonal damage^13^. Together, these studies indicate that oligodendrocyte protection would be clinically beneficial.

We have previously demonstrated that during inflammatory attack, oligodendrocyte death and demyelination is significantly exacerbated by genetic impairment of the integrated stress response (ISR)^14^, a cytoprotective mechanism that maintains cellular proteostasis. The ISR is triggered by stressors such as hypoxia/ischaemia, oxygen–glucose deprivation, viral infection, amino-acid starvation and endoplasmic reticulum (ER) stress^15^, in which accumulation of mis- or unfolded proteins threatens to overwhelm the cell’s secretory pathway^16^. This sensitivity makes the ISR particularly relevant in MS, as these stressors have all been implicated in MS lesion formation^10^.

The focal point of ISR induction is the phosphorylation of the α subunit of eukaryotic translation initiation factor 2 (eIF2α). During ER stress, for example, the kinase PERK phosphorylates eIF2α, resulting in inhibition of global protein synthesis to reduce the protein load on the ER and selective upregulation of the expression of transcription factors, such as ATF4, to activate a cytoprotective response. Once proteostasis is restored, protein synthesis is allowed to resume via a negative feedback loop, in which ATF4 initiates expression of CHOP, which in turn promotes expression of GADD34. GADD34 binds to protein phosphatase 1 (PP1), which dephosphorylates p-eIF2α to restore protein synthesis to its pre-stress levels^17^. If cellular stress remains unmitigated, however, the protective abilities of the ISR are overwhelmed. The ISR then induces apoptosis through the accumulation of CHOP, a pro-apoptotic protein^18^.

The ISR proteins CHOP, ATF4 and p-eIF2α have been found to be highly upregulated within MS and EAE lesions^19^,^20^,^21^,^22^. We hypothesized that ISR activation in these lesions was due to the high susceptibility of actively myelinating oligodendrocytes to ER stress^23^, as these cells are required to quickly produce vast amounts of plasma membrane and myelin proteins^24^. When faced with the additional stress of inflammatory attack, actively myelinating oligodendrocytes often undergo apoptosis^14^. Nevertheless, in genetically engineered mouse models, upregulation of protective ISR activity via increased expression of the eIF2α kinase PERK^25^ or inactivation of the eIF2α phosphatase component GADD34 (ref. 26) was found to significantly reduce the degree of oligodendrocyte loss and demyelination in the presence of inflammation. These genetic models provide proof of concept that enhancement of the ISR might provide protection to oligodendrocytes in inflammatory demyelinating disorders.

Guanabenz (2,6-dichlorobenzylidene aminoguanidine acetate) is an α2-adrenergic receptor agonist^27^ that has Food and Drug Administration (FDA) approval as an orally administered drug for hypertension. Nevertheless, a recent study demonstrated that guanabenz can also enhance protective ISR activity by inhibiting the binding of GADD34 to PP1, thereby inhibiting the dephosphorylation of p-eIF2α (ref. 28). Here we examine whether the ability of guanabenz to enhance the ISR could be used to pharmaceutically protect oligodendrocytes and myelin from inflammatory loss. To model inflammation, we use interferon-gamma (IFN-γ), a pro-inflammatory cytokine that has been strongly implicated in MS pathogenesis^29^,^30^. IFN-γ, normally undetectable in the CNS but measurable during the symptomatic phase of MS and EAE, has been shown to enhance inflammation in the CNS and exacerbate clinical symptoms in MS patients and EAE mice^31^,^32^,^33^.

In this study, we demonstrate that guanabenz protects oligodendrocytes both *in vitro* (in primary culture and cerebellar explants) and *in vivo* from IFN-γ-mediated death. Guanabenz also protects oligodendrocytes and ameliorates clinical symptoms in chronic EAE, with a concomitant decrease in the numbers of inflammatory CD4+ T cells within the CNS. Finally, treatment with guanabenz after development of clinical symptoms in relapsing-remitting EAE dampens the severity of the subsequent relapse. These data demonstrate the CNS-protective abilities during inflammation of an FDA-approved drug that enhances the ISR, a novel treatment strategy that may have potential as a therapeutic approach to treating MS.

## Results

### Guanabenz protects oligodendrocytes against IFN-γ

**Figure 1:**
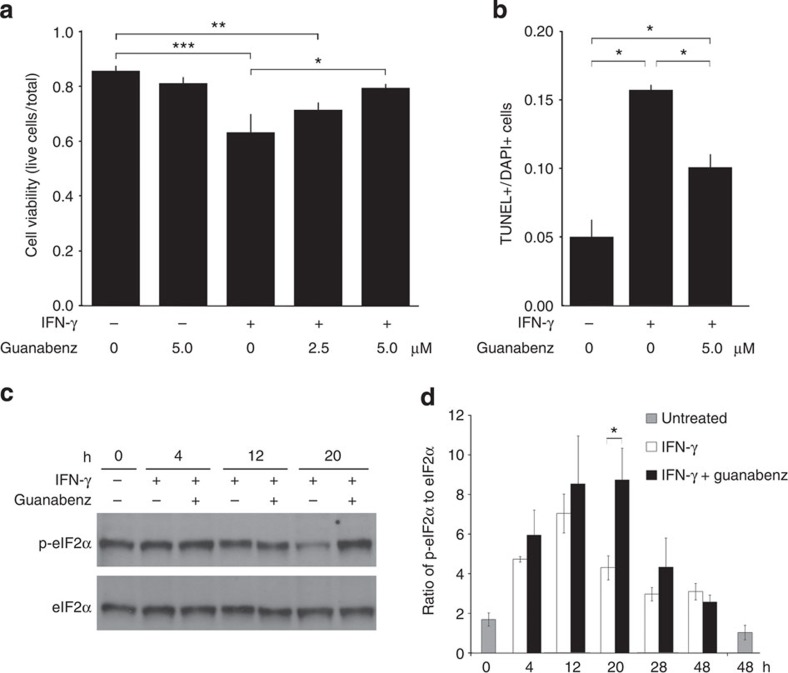
Guanabenz protects differentiated rat oligodendrocyte progenitor cells from IFN-γ-mediated death and prolongs the integrated stress response. (**a**) Quantification of dOPCs stained with PI/FDA to identify dead/live cells after 48 h of differentiation. Data represent three individual dOPC isolations, each with three replicates per group. (**b**) IFN-γ-mediated apoptotic death was confirmed via TUNEL staining of dOPCs 48 h after differentiation. *N*=3. (**c**) Western blot analysis of dOPCs treated continuously with IFN-γ alone or IFN-γ+5.0 μM guanabenz and probed with p-eIF2α, a marker of ISR activity, and eIF2α. (**d**) Quantification of extended time points in a western blot. Blot represents one of four individual isolations; graph represents the average of four isolations. Mixed results ANOVA (**a**), unpaired *t*-test (**b**,**d**), *P<0.05, **P<0.005, ***P<0.0005. Data are presented as mean±s.e.m.

We have previously reported that addition of IFN-γ to differentiating oligodendrocyte precursor cells (dOPCs) *in vitro* causes significant apoptotic cell death that is associated with ER stress^14^. We therefore sought to determine whether guanabenz could protect oligodendrocytes from IFN-γ-mediated death. We began by treating dOPCs with IFN-γ alone, guanabenz alone or IFN-γ plus guanabenz concomitantly for 48 h, at which point the ratio of live and dead cells in each group was measured. Whereas guanabenz treatment alone had no effect on cell survival, IFN-γ treatment resulted in a 22.5% decrease in dOPC survival compared with control untreated cells (Fig. 1a). Treatment with IFN-γ+2.5 μM guanabenz, however, significantly increased the number of surviving cells, and treatment with IFN-γ+5.0 μM guanabenz restored cell survival to control levels, demonstrating that guanabenz protected cells from IFN-γ-mediated death. TdT-mediated dUTP nick end labeling (TUNEL) staining confirmed that guanabenz protected the dOPCs from apoptotic death (Fig. 1b).

A recent study^28^ demonstrated that guanabenz protected HeLa cells from ER stress by inhibiting the dephosphorylation of eIF2α. We therefore examined p-eIF2α levels in dOPCs treated continuously with IFN-γ or IFN-γ+5.0 μM guanabenz over time by immunoblot. Expression of p-eIF2α increased steadily in both treatment groups as predicted, given that the ISR is activated in oligodendrocytes in response to the presence of IFN-γ (ref. 14). At 4 and 12 h, both groups showed similar levels of p-eIF2α expression (Fig. 1c,d). By 20 h, however, p-eIF2α expression had significantly decreased in dOPCs treated only with IFN-γ but remained high in cells treated with IFN-γ+5.0 μM guanabenz (*P*=0.043, unpaired *t*-test). This result suggests that guanabenz protected oligodendrocytes from IFN-γ-mediated apoptosis by driving persistence of the ISR. Interestingly, by 28 h after treatment (Fig. 1d), expression of p-eIF2α decreased in both groups and remained steady until 48 h, demonstrating that guanabenz’s enhancement of the ISR is transient *in vitro*.

### Guanabenz protects myelinating cerebellar slices from IFN-γ

**Figure 2:**
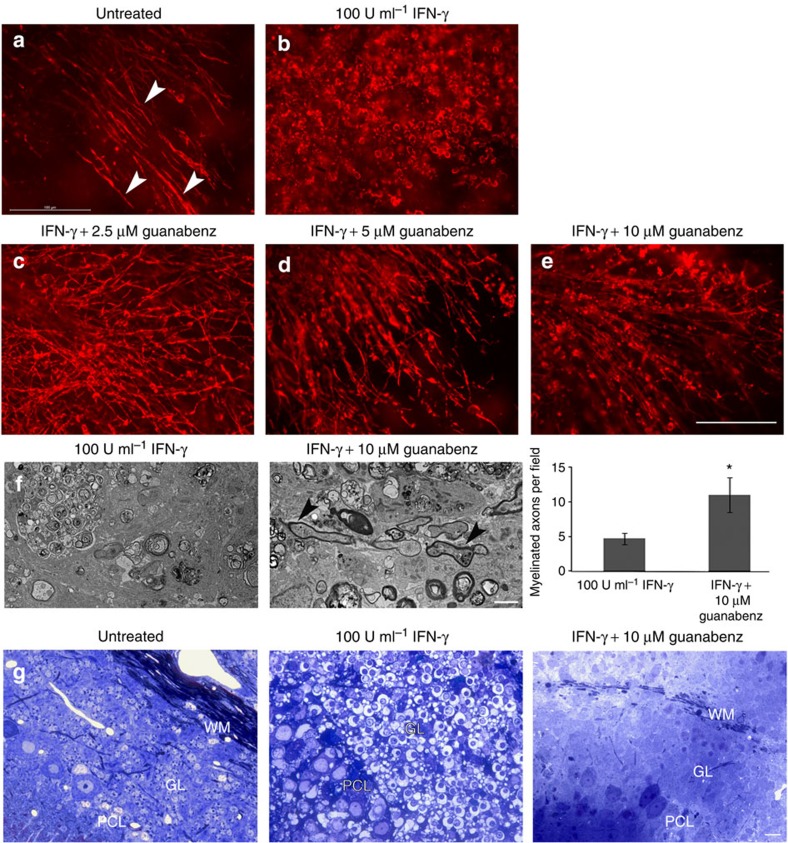
Guanabenz decreases IFN-γ-induced hypomyelination in rat cerebellar slice cultures. (**a**–**e**) Anti-MBP staining of myelinated fibres (**a**, arrowheads) in slice cultures that were (**a**) untreated, (**b**) treated with IFN-γ or (**c**–**e**) concomitantly treated with IFN-γ and 2.5, 5.0 or 10.0 μM guanabenz. Images representative of two or three slices per treatment; the experiment was performed twice. (**f**) Electron microscopy analysis of slice cultures. Note the significant increase in the number of myelinated axons (arrowheads) when guanabenz and IFN-γ were concomitantly added to slices. Myelinated axons per field were determined by analysis of a minimum of 200 axons per condition. (**g**) Toluidine blue staining of slices left untreated, treated with IFN-γ alone or treated with IFN-γ and guanabenz. Images represent a minimum of three sections per treatment. Unpaired *t*-test, *P<0.05. Data are presented as mean±s.e.m. Scale bars, 100 μm (**a**–**e**), 2 μm (**f**) and 20 μm (**g**). WM: white matter, GL: granule cell layer, PCL: Purkinje cell layer.

Having found that guanabenz is able to protect dOPCs from IFN-γ-mediated death, we next investigated whether the surviving dOPCs were able to successfully myelinate axons. We treated rat cerebellar slice cultures sectioned at postnatal day 11 (P11) for 7 days with IFN-γ alone or IFN-γ and increasing doses of guanabenz. In this experimental time frame, dOPCs are able to mature and begin to myelinate axons, as demonstrated by the finding that myelin basic protein (MBP) immunostaining in normal explants at P20 show distinct and well-organized myelinated fibres (Fig. 2a, arrowheads). When IFN-γ was added to these slice cultures, however, myelin organization was severely disrupted (Fig. 2b). Co-culture with 2.5, 5.0 or 10.0 μM guanabenz resulted in restored myelination to IFN-γ-treated explants (Fig. 2c–e).

Electron microscopy analysis to examine the extent of myelination in the explants revealed that, compared with sections treated only with IFN-γ, sections treated concomitantly with guanabenz had 57.5% more myelinated axons (*P*=0.022, unpaired *t-*test; Fig. 2f, arrowheads). Indeed, toluidine blue staining revealed that the IFN-γ treatment alone appeared to destroy the general cytoarchitecture of the slice tissue (Fig. 2g), whereas addition of 10.0 μM of guanabenz along with IFN-γ protected the tissue such that myelination and the cellular architecture were preserved. Therefore, guanabenz treatment appears not only to reduce IFN-γ-mediated hypomyelination but also to have neuroprotective properties as well.

### Guanabenz protects oligodendrocytes from IFN-γ *in vivo*

We have previously described a transgenic mouse model system in which the ectopic expression of IFN-γ by astrocytes is regulated by tetracycline^14^. In these mice, the transcriptional control region of the glial fibrillary acidic protein (GFAP) gene drives expression of the tTA protein, which is the transcriptional activator of the tetracycline regulatory element (TRE) that drives IFN-γ expression. Transcriptional activation of the *TRE/IFN-γ* transgene by tTA is prevented by the presence of doxycycline, a tetracycline derivative. This inducible system allows for regulated expression of IFN-γ specifically in the CNS and independently from the adaptive immune response. Using these transgenic mice, we have shown that CNS-specific expression of IFN-γ results in significant oligodendrocyte loss and hypomyelination in the absence of an adaptive immune response^14^. Moreover, studies of *GFAP/tTA;TRE/IFN-γ* transgenic mice revealed that IFN-γ-mediated insults in the CNS became more severe when ISR capabilities were diminished^14^, and less severe when the ISR was enhanced^26^. We hence used these mice to investigate whether guanabenz could protect oligodendrocytes from inflammation-induced cell death and prevent myelin loss *in vivo*.

**Figure 3:**
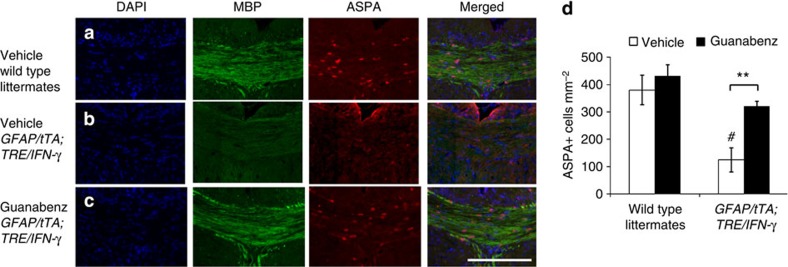
Guanabenz protects oligodendrocytes and myelin from IFN-γ-mediated loss *in vivo*. (**a**–**c**) Immunofluorescent staining for MBP and ASPA, a mature oligodendrocyte marker, in the medial corpus callosum of (**a**) vehicle-treated wild-type littermates, (**b**) vehicle-treated *GFAP/tTA;TRE-IFN-γ* transgenic mice and (**c**) guanabenz-treated *GFAP/tTA;TRE-IFN-γ* mice at P18. Mice were treated with vehicle or 4 mg kg^−1^ of guanabenz daily from P7 to P18. (**d**) Quantification of ASPA+ cells in the medial corpus callosum of wild type littermates and *GFAP/tTA;TRE-IFN-γ* mice treated with vehicle or guanabenz. Images represent four to six mice per group; graph represents the average of values from four to six mice per group. Unpaired *t*-test, **P<0.005, ^#^*P*<0.05 as compared with vehicle-treated control. Scale bar, 200 μm. Data are presented as mean±s.e.m.

In wild type littermates, immunostaining at P18 for MBP and aspartoacylase (ASPA), a mature oligodendrocyte marker^34^,^35^, revealed distinct myelin tracts and a large population of mature oligodendrocytes in the corpus callosum (Fig. 3a), whereas in vehicle-treated mice with CNS-specific expression of IFN-γ, a 60.6% (*P*=0.011, unpaired *t-*test) loss of mature oligodendrocytes and hypomyelination were observed (Fig. 3b). IFN-γ-expressing mice that were treated daily from P7 with 4 mg kg^−1^ of guanabenz showed a 51.3% (*P*=0.008, unpaired *t*-test) increase in the number of mature oligodendrocytes compared with vehicle-treated mice and a restoration of myelination to levels observed in wild type littermates (Fig. 3c,d). These findings demonstrate that guanabenz is able to protect oligodendrocytes and myelin from the detrimental effects of IFN-γ *in vivo.*

### Guanabenz ameliorates chronic EAE disease symptoms in mice

EAE is a CD4+ T cell-mediated model of MS in which adult mice are immunized with a component of myelin in adjuvant (typically complete Freund’s adjuvant, CFA). The resulting myelin peptide-activated CD4+ T cells infiltrate the CNS, resulting in lesioned areas of inflammation, oligodendrocyte apoptosis, demyelination and axonal degeneration predominantly in the spinal cord. Inflammatory insults in mice with EAE manifest as MS-like clinical symptoms such as ataxia and paralysis^36^. As we clearly observed the ability of guanabenz to protect developing oligodendrocytes and myelination from IFN-γ-induced loss *in vitro* and *in vivo,* we next sought to explore whether guanabenz treatment could protect mature oligodendrocytes and myelin to provide therapeutic benefit to mice with chronic EAE.

We began by determining a dose of guanabenz that could potentially enhance ISR activity in EAE mice. Data from our dOPC cultures (Fig. 1) and cerebellar explants (Fig. 2) demonstrated that 2.5–10.0 μM guanabenz was sufficient to protect oligodendrocytes and myelin from inflammatory loss. Pharmacokinetic analysis of serum and brain tissue from wild type EAE mice treated with 4, 8 or 16 mg kg^−1^ of guanabenz daily for >20 days revealed a striking concordance between these efficacious *in vitro* concentrations and the EAE brain exposures (Supplementary Table 1): brain tissue levels of drug were above 2 μM at 2 h in all dosage groups, and were above 2 μM at 4 h for the 8 and 16 mg kg^−1^ groups. Guanabenz levels were also substantially lower in serum than in the brain, with the brain:serum ratio of guanabenz in our EAE mice similar to those reported in rats^37^ and rhesus monkeys^38^. While guanabenz was eliminated much more rapidly in our mice than as reported in humans, the serum exposure in mice 4 h post injection was similar to that previously reported in human volunteers at doses that have been approved by the FDA for therapeutic use^39^. These findings indicated that 4–16 mg kg^−1^ treatment with guanabenz in mice would likely achieve CNS levels sufficient to modulate the ISR.

**Figure 4:**
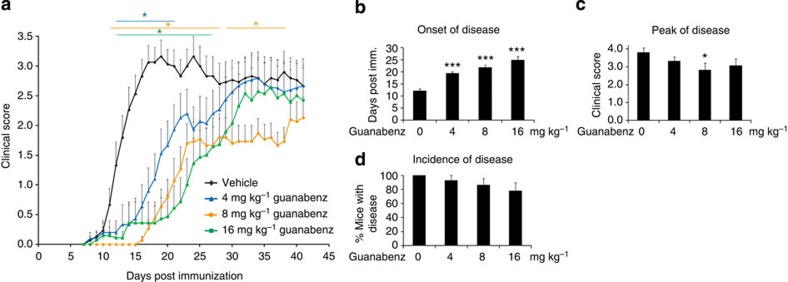
Guanabenz treatment delays and alleviates clinical symptoms in mice with chronic EAE. (**a**) Clinical scores of wild type C57BL/6J female mice immunized with CFA and MOG_35–55_ to induce chronic EAE, treated with vehicle (*n*=15) or 4 mg kg^−1^ (*n*=15), 8 mg kg^−1^ (*n*=15) or 16 mg kg^−1^ (*n*=14) guanabenz daily from PID7 to the end of the study. (**b**–**d**) Average onset of disease, defined as the day a clinical score of 1.0 was first reached in each mouse (**b**), peak of disease (**c**) and incidence of disease (**d**) of all treatment groups. Data in (**a**–**d**) represent one of two studies conducted with similar results and presented as mean±s.e.m. Unpaired *t-*test, *P<0.05, ***P<0.0005 compared with vehicle.

Indeed, while vehicle-treated EAE mice typically develop clinical symptoms about 10 days after immunization with disease severity peaking roughly a week later (Fig. 4a), daily treatment of EAE mice with 4, 8 or 16 mg kg^−1^ of guanabenz beginning at post-immunization day 7 (PID7) significantly delayed the onset of clinical symptoms (defined as a clinical score of 1.0; Fig. 4a,b). Notably, the average peak of clinical disease in EAE mice treated with 8 mg kg^−1^ guanabenz (2.8) was also significantly lower than in vehicle-treated EAE mice (3.8, *P*=0.032, unpaired *t*-test), such that the average vehicle-treated EAE mouse developed hindlimb paralysis and even forelimb paresis, whereas the average guanabenz-treated EAE mouse experienced only hindlimb paresis (Fig. 4c). In addition, the incidence of disease in all guanabenz-treated EAE mice was lower than in vehicle-treated EAE mice (Fig. 4d). These results demonstrate that guanabenz treatment significantly delays disease onset at all doses tested, while treatment with 8 mg/kg guanabenz also diminishes severity of disease.

### Guanabenz alters the molecular and cellular response to EAE

**Figure 5:**
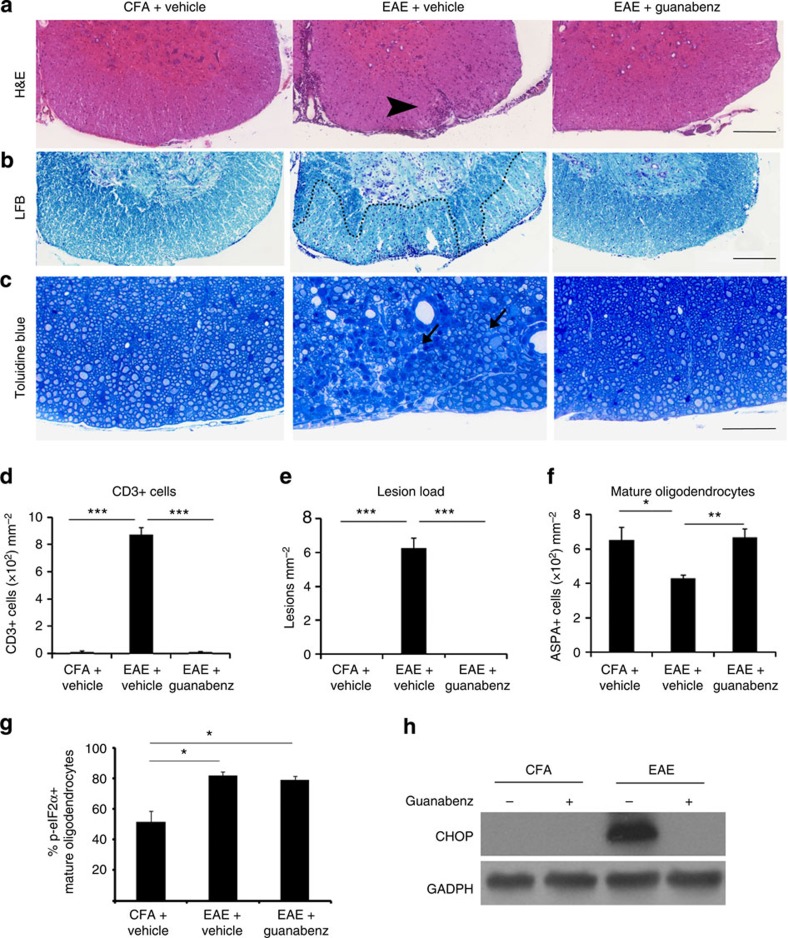
Guanabenz treatment alters T cell distribution and upregulates ISR activity while protecting oligodendrocytes in chronic EAE. All analyses conducted using lumbar spinal cords of PID15 mice immunized with adjuvant only (CFA) or adjuvant with MOG_35–55_ (EAE), then treated with vehicle or guanabenz from PID7 to 15. (**a**) Haematoxylin and eosine (H&E) staining. Note only the vehicle-treated EAE sample displays cellular infiltrates (arrowhead). (**b**) LFB staining. Note the demyelinated focal areas (dotted areas) in vehicle-treated EAE mice only. (**c**) Higher magnification of sections stained with toluidine blue. Note the demyelination (arrows) in areas of cellular infiltration in vehicle-treated EAE mice. (**d**) Quantification of cells positive for CD3, a T cell marker. (**e**) Isolated pockets of these CD3+ areas were considered ‘lesion’ areas and quantified. (**f**) Quantification of cells positive for ASPA, a mature oligodendrocyte marker, in ‘lesion’ areas. (**g**) Quantification of cells positive for both p-eIF2α and tubulin polymerization promoting protein (TPPP), a mature oligodendrocyte marker. Unpaired *t-*test, *P<0.05, **P<0.005, ***P<0.0005. Data in **d**–**g** represent an average of five to six mice per group, presented as mean±s.e.m. (**h**) Immunoblot analysis of the pro-apoptotic ISR protein CHOP in lumbar spinal cord lysates. GAPDH is presented as a loading control. Blot representative of *n*=4 per group. Scale bars, 200 μm (**a**,**b**), 50 μm (**c**).

Given the ability of guanabenz treatment to significantly ameliorate EAE, we next sought to determine the underlying mechanism of this protection. Lumbar spinal cords were isolated from PID15 EAE mice (age corresponding to the average peak of disease in vehicle-treated EAE mice) that were treated daily with vehicle or 8 mg kg^−1^ of guanabenz beginning PID7 for immunohistochemical and biochemical analyses. Since EAE and MS are characterized by focal inflammatory lesions of demyelination and oligodendrocyte loss, we analysed the ability of guanabenz treatment to decrease the number of spinal cord lesions as well as the degree of oligodendrocyte loss within the lesions. Histological analysis of lumbar spinal cord cross-sections revealed inflammatory foci in the vehicle-treated EAE sections as expected (Fig. 5a, arrowhead), but surprisingly none in the guanabenz-treated EAE tissue. Staining with luxol fast blue (LFB) also revealed demyelination around these areas of cell infiltration (Fig. 5b, dotted areas) in vehicle-treated EAE tissue only. Both findings were further emphasized in higher magnification images of toluidine blue-stained sections (Fig. 5c, arrows). To identify and analyse these focal areas of infiltration, we used CD3 as a general T cell marker and confirmed that mice immunized with adjuvant only, which did not develop any clinical symptoms, also did not have any detectable CD3+ cells in the spinal cord as expected (Fig. 5d, Supplementary Fig. 1). In vehicle-treated EAE mice, however, we identified large focal areas in the white matter that contained on average 800 CD3+ cells per mm^2^. Designating these highly concentrated areas of CD3+ cells as ‘lesion’ sites (Fig. 5e), we found that the vehicle-treated EAE mice had 65.7% (*P*=0.026, unpaired *t*-test) of the mature oligodendrocytes found in regions of matched size and location in mice immunized with adjuvant only (Fig. 5f). Anti-CD3 immunostaining in the guanabenz-treated EAE sections, however, revealed no detectable CD3+ cells and hence no lesion sites, and indeed counts of mature oligodendrocytes in regions of matched size and location to vehicle-treated EAE mice revealed cell numbers comparable to those in mice treated with adjuvant only (Fig. 5d–f). Thus, at PID15, the typical EAE characteristics of infiltrating T cells within the spinal cord, oligodendrocyte loss and regions of demyelination are present in vehicle-treated but not guanabenz-treated mice.

The ongoing presence of T cells in the spinal cord at this EAE time point is heavily dependent on debris resulting from oligodendrocyte and myelin loss^40^. As our earlier *in vitro* and *in vivo* experiments had demonstrated that enhancement of the ISR with guanabenz treatment can protect oligodendrocytes from death, we next examined whether the ISR could be playing a role in delaying and alleviating EAE by protecting mature oligodendrocytes from inflammatory loss, thereby limiting detectable T cell presence at PID15. We therefore stained PID15 EAE lumbar spinal cord tissue sections with markers for p-eIF2α and mature oligodendrocytes. These data showed that, while roughly 50% of the mature oligodendrocytes in adjuvant-only-treated mice expressed p-eIF2α, more than 80% of mature oligodendrocytes expressed p-eIF2α in both the vehicle- and guanabenz-treated EAE mice (Fig. 5g). As MS and EAE lesions^19^,^20^,^21^,^22^ have previously been shown to have high ISR activity, the finding that vehicle-treated EAE tissue had high expression of the ISR pathway was expected. Since sections from guanabenz-treated EAE mice appeared similarly lesion-free and intact as those from adjuvant-only-treated mice, however, the finding that the ISR was also highly upregulated in the guanabenz-treated sections suggests that the ISR may have a role in enhancing oligodendrocyte survival and subsequently delaying EAE onset and alleviating disease severity.

In PID15 EAE lumbar spinal cord lysates, immunoblots probing the protein CHOP revealed further evidence of ISR activity (Fig. 5h). CHOP is a pro-apoptotic protein that is downstream of p-eIF2α in the ISR pathway, and its high expression is indicative of cell loss as a result of an overwhelmed ISR. Interestingly, Tsaytler *et al.*^28^ found that as guanabenz merely prolonged the phosphorylation of eIF2α, treatment with guanabenz did not induce higher CHOP expression. We found that CHOP was highly expressed in vehicle-treated EAE tissue, but not in guanabenz-treated EAE tissue or samples from mice immunized with adjuvant only (Fig. 5h). Taken together, these findings of the key markers of ISR activity, p-eIF2α and CHOP, indicate that guanabenz treatment enhanced the ISR in EAE mice, potentially resulting in ISR-mediated protection of oligodendrocytes from EAE inflammatory loss and subsequently inhibiting continuation of the activated immune response within the CNS.

### Guanabenz alters the number of activated CD4+ cells in EAE

**Figure 6:**
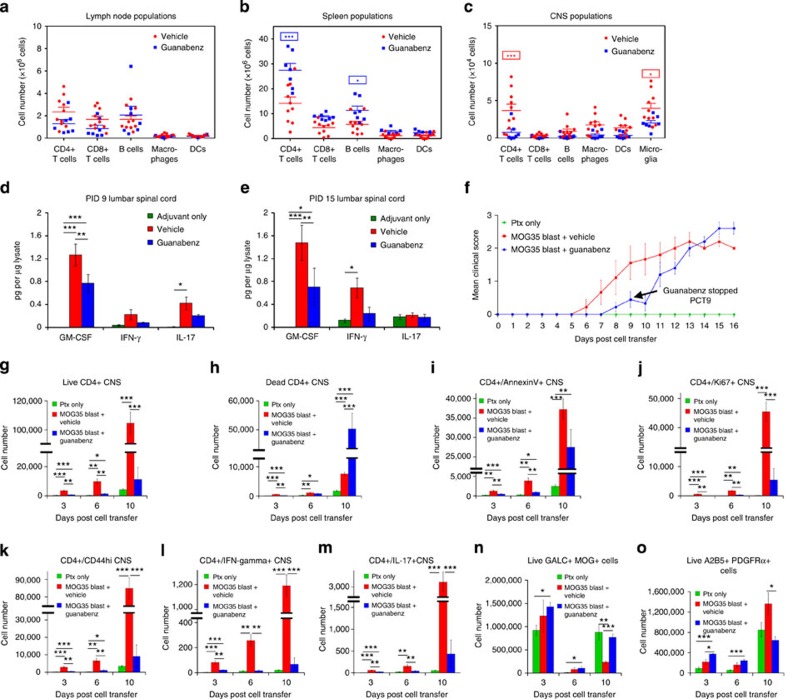
Guanabenz treatment protects oligodendrocytes and alters CD4+ T cell populations in two EAE mouse models. Flow cytometry analysis of immune cell populations in the (**a**) inguinal lymph nodes, (**b**) spleen and (**c**) CNS, taken from PID15 mice with actively induced chronic EAE treated daily with vehicle or guanabenz beginning PID7. Data are representative of four mice per group; experiment performed twice. (**d**,**e**) Analysis of IFN-γ and IL-17 protein expression in the lumbar spinal cord of PID9 and PID15 chronic EAE mice. PID9, *n*=4–5 mice per group, PID15, *n*=6–9 mice per group. (**f**–**o**) Flow cytometry analysis of adoptive transfer EAE mice. Recipient mice were treated daily with vehicle or guanabenz beginning PCT day 0. C57BL/6 mice that received no blast cells and only two pertussis toxin treatments (Ptx only) were included as flow analysis controls. (**f**) All mice were followed for EAE disease severity until euthanized, with five mice in each group remaining on day 16. The last guanabenz treatment was on day 9, and the subsequent rapid clinical decline of these animals demonstrated that they were the recipients of active T cells and the protective nature of guanabenz. On days 3, 6 and 10 the CNS was collected from four representative mice in each treatment group, and the number of (**g**) total live CD4+ T cells, (**h**) dead CD4+ T cells, (**i**) AnnexinV+ CD4+ T cells, (**j**) Ki67+ CD4+ T cells, (**k**) CD44hi CD4+ T cells, (**l**) IFN-γ+ CD4+ Th1 cells and (**m**) IL-17+ CD4+ Th17 cells was assessed via flow cytometry. The number of (**n**) live mature GALC+ MOG+ oligodendrocytes present within the CNS and the number of (**o**) A2B5+ PDGFRα+ early progenitor OPCs were also assessed via flow cytometry. The data are presented as the average number of cells over time. *P<0.05, **P<0.005, ***P<0.0005, as compared with vehicle-treated mice. Data represents average of four mice per group, presented as mean±s.e.m.

While our earlier experiments clearly show that enhancement of ISR activity can protect oligodendrocytes and myelin, upregulation of this pathway has been found to lead to suppression of the inflammatory response^16^,^41^,^42^. Studies have also shown that agonism of the α2-adrenergic receptor, another known function of guanabenz, may also modulate inflammation^43^,^44^,^45^. We therefore tested whether guanabenz treatment had any immunomodulatory effects in PID15 EAE mice. Compared with vehicle-treated EAE mice, EAE mice treated daily from PID7 to PID15 with 8 mg kg^−1^ guanabenz showed no difference in the numbers of CD4+ T cells, CD8+ T cells, B cells, macrophages or dendritic cells in the lymph node as determined by flow cytometric analyses (Fig. 6a). In addition, guanabenz treatment had no effect on lymph node T cell proliferation or Th1 (IFN-γ) or Th17 (interleukin (IL)-17 and granulocyte–macrophage colony-stimulating factor (GM-CSF)) cytokine production in response to anti-CD3 (1 μg ml^−1^), OVA_323–339_ or myelin oligodendrocyte glycoprotein (MOG) 35–55 (20 μg ml^−1^; Supplementary Fig. 2a–d). By contrast, guanabenz-treated EAE mice showed a significant increase in the number of splenic CD4+ T cells and B cells (Fig. 6b). When equal numbers of total splenocytes were reactivated *ex vivo*, however, again no significant differences in recall responses were found (Supplementary Fig. 2e–h). Together, the above findings suggest that guanabenz treatment during EAE has no effect on peripheral T cell proliferation or cytokine production, but may cause retention of CD4+ T cells within the spleen.

In the CNS, in line with our histological findings (Fig. 5), significantly fewer CD4+ T cells and microglia were found in the guanabenz-treated EAE mice (Fig. 6c). While ISR-mediated protection of oligodendrocytes may limit subsequent T cell responses, it is also possible that guanabenz treatment directly affects the ability of CD4+ T cells to traffic into the CNS and/or become fully activated. We began our examination of these possibilities by evaluating cytokine expression at the initiation (PID9) and peak (PID14–15) of clinical disease in the lumbar spinal cord tissue. Interestingly, at both PID9 and PID15, protein levels of GM-CSF in the vehicle- and guanabenz-treated EAE mice were significantly higher than those in mice treated with adjuvant only (Fig. 6d,e). Similarly, vehicle- and guanabenz-treated EAE mice yielded higher protein levels of IFN-γ than adjuvant mice at both time points, which was reflected in higher IFN-γ mRNA levels (Supplementary Fig. 3). While this also held true for IL-17 protein and mRNA levels at PID9, both protein and mRNA levels of IL-17 were similar in all samples by PID15 (Fig. 6d,e, Supplementary Fig. 3). These findings indicate that guanabenz treatment is not inhibiting the presence of the key encephalitogenic factors needed for EAE development within the CNS.

We explored the effect of guanabenz on T cell trafficking into the CNS further using the MOG_35–55_ adoptive transfer model of EAE in C57BL/6 mice. MOG_35–55_ blast cells were transferred into naïve recipient mice that then received daily treatment of either vehicle or guananbenz (8 mg kg^−1^). The mice were monitored for disease over a 16-day-time course, and the data show that clinical disease severity peaked at a clinical score of 2 in the vehicle-treated mice and was strikingly reduced in the guanabenz-treated mice (Fig. 6f).

The use of the adoptive transfer model guaranteed that guanabenz treatment could not affect initial activation or expansion of T cells while allowing modulation of the *in vivo* reactivation and subsequent proliferation of the myelin peptide-specific CD4+ T cells required for transfer EAE^46^. To determine how guanabenz treatment might alter CD4+ T cell activation *in vivo*, spleen and CNS samples were collected 3, 6 and 10 days post-cell transfer (PCT). Vehicle- and guanabenz-treated recipient mice both had significantly higher numbers of live CD4+ T cells within the CNS on PCT3 and PCT6 compared with non-recipient mice injected with pertussis only, indicating that the drug does not prevent initial T cell infiltration into the CNS. On PCT10, however, vehicle-treated recipients displayed a significant increase in the number of live CD4+ T cells compared with earlier time points, while guanabenz-treated recipient mice only displayed a minimal increase (Fig. 6g). These data correlate with our findings in the actively-induced chronic EAE model, in which guanabenz-treated mice had significantly lower CD4+ T cell numbers compared with vehicle-treated mice (Fig. 6c). Vehicle-treated mice on PCT10 also displayed significantly greater numbers of proliferating (Ki67+), activated (CD44hi) Th1 (IFN-γ+) and Th17 (IL-17+) CD4+ T cells within the CNS as compared with guanabenz-treated mice (Fig. 6j–m and Supplementary Figs 5–8). Meanwhile, in the spleen, vehicle-treated mice showed an increase in numbers of Th1 and Th17 cells from PCT3 to PCT10 (Supplementary Figs 4–8). Conversely, the guanabenz-treated PCT10 mice had significantly higher numbers of dead CD4+ T cells in the CNS as compared with vehicle-treated PCT10 mice (Fig. 6h,i) and significantly decreased numbers of live CD4+ T cells (Fig. 6g), resulting in significantly lower numbers of Ki67+, CD44hi, IFN-γ+ and IL-17+ CD4+ T cells (Fig. 6j–m). Guanabenz treatment also resulted in an increase in the number of dead CD4+ T cells within the spleen on PCT3 compared with vehicle treatment (Supplementary Figs. 4-8).

We also used flow cytometry^47^ to characterize oligodendrocyte lineage cells in the adoptive transfer recipients. Consistent with our histological findings (Fig. 5f), there was not an observed loss of mature (GALC+, MOG+) oligodendrocytes in the guanabenz-treated mice over the 10-day-time course, while this was readily observed in the vehicle-treated recipients (Fig. 6n, Supplementary Figs 9 and 10). The loss of oligodendrocytes in the vehicle-treated recipients correlated with an increase in oligodendrocyte progenitor cells (A2B5+, PDGFRα+), which was not observed in the guanabenz-treated recipients (Fig. 6o).

Collectively, these data demonstrate that guanabenz treatment results in both a decrease in the number of activated CD4+ T cells and an increase in the number of dead CD4+ T cells in the CNS while protecting mature oligodendrocytes. Whether the decreased T cell activation occurs directly by a T cell- or CNS antigen-presenting cell (APC)-intrinsic mechanism and/or indirectly by limiting the amount of myelin antigen available secondary to guanabenz-induced oligodendrocyte protection requires additional investigation.

### Guanabenz alleviates relapse in relapsing-remitting EAE

**Figure 7:**
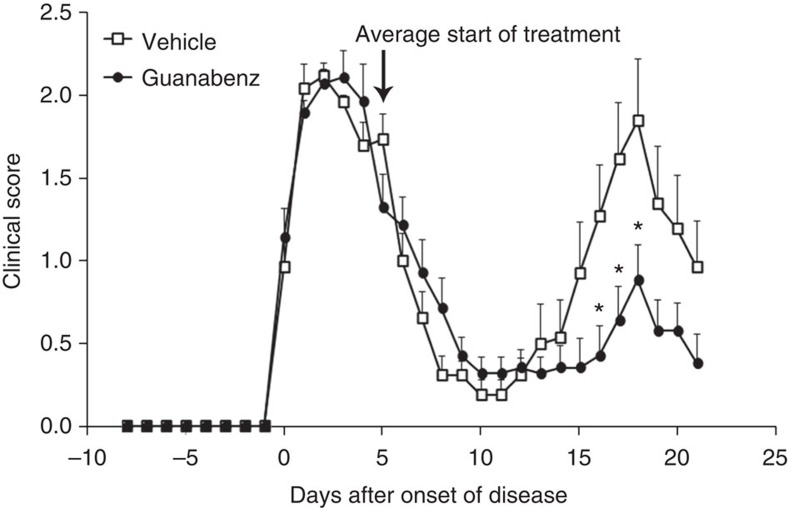
Guanabenz treatment alleviates clinical severity of relapse in mice with relapsing-remitting EAE. Six- to eight-week old female SJL mice were immunized with proteolipid protein 139–151 to induce R-EAE. R-EAE mice that displayed an acute phase of disease were treated at the beginning of remission (on average 5 days after onset of disease) with vehicle (*n*=13) or 8 mg kg^−1^ of guanabenz (*n*=14) daily and monitored for clinical symptoms. All R-EAE mice that displayed no relapse (9 out of 22 vehicle-treated and 9 out of 23 guanabenz-treated) were removed from the analysis. Unpaired *t-*test, *P<0.05. Data are presented as mean±s.e.m. Clinical scores of each treatment group are aligned by day of onset of disease (defined as the day the mouse reaches a clinical score of 1) and averaged by day. One representative experiment of two is presented.

The ability of guanabenz to protect oligodendrocytes and myelin both *in vitro* and *in vivo,* in addition to delaying and alleviating disease in chronic EAE, suggests that it has potential as a therapeutic agent for MS. In patients with relapsing-remitting MS, the predominant human disease presentation, treatment is initiated only when clinical symptoms are already present. To investigate whether guanabenz has therapeutic potential for this clinical setting, we used the SJL/J mouse relapsing-remitting EAE (R-EAE) model^48^,^49^. Similar to human patients with MS, these mice develop symptoms, undergo remission and experience subsequent clinical relapses. We thus treated SJL/J mice with R-EAE with 8 mg kg^−1^ of guanabenz daily beginning at the onset of remission. Encouragingly, guanabenz-treated R-EAE mice displayed a 47.9% reduction in clinical severity at peak of relapse (clinical score of 0.9) compared with vehicle-treated R-EAE mice (1.8, *P*=0.038, unpaired *t*-test, Fig. 7), such that guanabenz-treated R-EAE mice experienced tail limpness, the initial sign of the clinical onset of EAE, whereas vehicle-treated R-EAE mice showed hindlimb ataxia. These results indicate that treatment with guanabenz can be effective following the onset of disease.

## Discussion

We demonstrated that guanabenz, an FDA-approved oral drug that enhances the ISR, effectively protected oligodendrocytes from inflammatory cytokine-mediated death in primary culture, in cerebellar explants and *in vivo*. Furthermore, treatment with guanabenz dampened and delayed clinical disease in chronic EAE mice and ameliorated relapse severity in relapsing-remitting EAE mice. Together, these studies attest to the potential of ISR enhancement in protecting oligodendrocytes and myelin against inflammation-mediated loss, and provide support for guanabenz as a pharmacological candidate for achieving this effect.

Enhancement of ISR activity, in addition to protecting oligodendrocytes and myelin, has been found to be closely linked to a diminished inflammatory response^16^,^41^,^42^. We demonstrated that guanabenz has oligodendrocyte-protective capabilities that are independent of its potential immunomodulatory effects: drug treatment *in vitro* and *in vivo* (using primary oligodendrocytes, cerebellar slices and *GFAP/tTA; TRE/IFN-γ* transgenic mice) protected oligodendrocytes against the effects of IFN-γ despite the lack of adaptive immune cells in these models. To diminish the effects that guanabenz might have on the generation and function of the inflammatory response that characterizes EAE, we initiated treatment of chronic EAE mice at 7 days post immunization, at which point the first round of MOG_35–55_-specific CD4+ T cells present within the draining lymph nodes have been activated^36^. This treatment scheme resulted in significant delay of clinical disease onset and alleviation of disease symptoms, with no significant effect on proliferation or cytokine production of splenic and lymph node T cells, indicating that peripheral immune cells maintained function. In addition, adoptive transfer EAE experiments revealed that guanabenz did not affect the initial infiltration of activated T cells into the CNS. Nevertheless, compared with vehicle-treated chronic EAE, the significant decrease in T cells in the guanabenz-treated chronic EAE mouse CNS 15 days after immunization signified that drug treatment did indeed alter the inflammatory response.

During inflammation, the infiltration and sustained presence of activated T cells in the CNS requires a cascade of specific events that are influenced by the interaction of the immune cells with the CNS environment^46^. For example, CD4+ T cells need to interact with cognate peptide being presented by APCs to be retained within the target tissue and must also receive sufficient restimulation within target tissue to avoid undergoing apoptosis^40^. We have demonstrated here that guanabenz-mediated enhancement of the ISR is sufficient to protect oligodendrocytes from inflammation-induced loss, a state that would result in a reduction of the apoptotic cell and myelin debris required for maintaining an inflammatory response, which could explain the decreased number of CD4+ T cells present within the CNS in the guanabenz-treated mice. In support of this possibility, the genetic protection of oligodendrocytes from apoptosis has been shown to result in a dramatic inhibition of clinical symptoms and, importantly, the inflammatory response in EAE^13^. Although our data indicate that guanabenz does not prevent the initial infiltration of CD4+ T cells into the CNS 3 and 6 days after encephalitogenic T cell transfer, a significantly lower number of CD4+ Th1 and Th17 effector T cells are present within the CNS of guanabenz-treated recipient mice 10 days post transfer. In addition, other aspects of T cell function might be altered by the presence of the drug. The ISR is known to affect T cell cytokine production^50^, such that sustained activation of the ISR in the presence of guanabenz could help explain the noted alteration to T cell activity in the treated animals. Moreover, independent from its ISR effects, guanabenz is also an α2-adrenergic receptor agonist, and other drugs with similar activity have been demonstrated to modulate the inflammatory response^51^,^52^. The full effects of guanabenz on the clinical and histopathologic response of EAE are likely a combination of its oligodendrocyte-protective effects in combination with modest immunomodulatory activities.

Our results demonstrate the considerable protective effects of guanabenz treatment on oligodendrocytes and myelin in the presence of inflammation. In addition, developing cerebellar slice cultures treated with IFN-γ displayed a dramatic disruption in cytoarchitecture that was ameliorated by guanabenz treatment, suggesting that the enhancement of the ISR might also provide neuroprotection in the face of inflammation. This possibility is deserving of further study.

Sustained translational repression, due to prolonged eIF2α phosphorylation, could prove detrimental to myelinating cells. Nevertheless, guanabenz prolongs stress-induced eIF2α phosphorylation by disrupting GADD34–PP1R15A interaction, while sparing non-stress-related CReP/PP1R15B p-eIF2α phosphatase activity^28^. This characteristic of guanabenz avoids the problems associated with salubrinal, another ISR-enhancing drug^26^,^53^, which inhibits the activity of both p-eIF2α phosphatase complexes. Moreover, our pharmacokinetic data indicated that guanabenz is cleared from the brain within 12 h of administering a 16-mg kg^−1^ dose, suggesting that the inhibition of p-eIF2α dephosphorylation is transient, even in animals treated with the highest doses used here. Furthermore, GADD34 mutant animals, which completely lack the GADD34–PP1R15A activity, myelinate normally even in the presence of persistent inflammation^26^, indicating that transient pharmacological inhibition of GADD34 activity would likely be well tolerated by mature oligodendrocytes and beneficial to remyelinating oligodendrocytes in an inflammatory environment.

Various cytotoxic events activate the ISR leading to eIF2α phosphorylation, whereas unstressed cells do not display elevated levels of p-eIF2α. Therefore, the inhibition of GADD34-mediated eIF2α dephosphorylation should have minimal impact, if any, on healthy cells. In support of this premise, unstressed GADD34 −/− mutant cells display normal levels of p-eIF2α *in vitro*^54^ and *in vivo* (Supplementary Fig. 11); similarly, unstressed guanabenz-treated cells display normal translational activity *in vitro*^28^ and have normal p-eIF2α levels *in vivo* (Supplementary Fig. 11). Therefore, a therapeutic approach that targets the inhibition of GADD34-mediated eIF2α dephosphorylation should display selectivity for only those cells experiencing a cytotoxic event, diminishing the possibility of side effects.

Although this study does not directly address the impact of an enhanced ISR response on remyelination, our data demonstrate the protective effect of guanabenz on myelinating oligodendrocytes, *in vitro* and *in vivo*, in the presence of inflammation. We are currently addressing the effect of guanabenz on models more suitable for a direct assessment of remyelination. Nevertheless, even if the enhancement of the ISR only provides protection to mature oligodendrocytes against inflammation, thereby diminishing demyelination, it would likely still have significant therapeutic value.

In conclusion, our studies have demonstrated that pharmaceutical enhancement of the ISR is effective in protecting oligodendrocytes and myelin from inflammatory-mediated destruction. Treatment with guanabenz, an FDA-approved, orally administered agent, significantly dampens and delays disease symptoms in mouse models of MS. Moreover, the quantities used in these studies provide serum drug levels that have been safely achieved in human subjects using FDA-approved dosages^39^. These results provide support for guanabenz as the first oligodendrocyte-protective agent for the alleviation of inflammatory-mediated demyelination in diseases such as MS.

## Methods

### Guanabenz

Guanabenz was purchased from MP Biomedicals (no. 193657), solubilized to 5 mg ml^−1^ in dH_2_O, and aliquoted and stored at −20 °C. Immediately before use, aliquots were thawed and diluted to desired concentrations in media for *in vitro* treatments and in sterile 0.9% NaCl for *in vivo* treatments.

### Isolation and treatment of oligodendrocyte precursor cells

OPCs were isolated and purified to >95% homogeneity from cortices of 6- to 7-day-old Sprague–Dawley rats by immunopanning as previously described^55^. Briefly, cortices were extracted, diced and digested with papain at 37 °C. Cells were then triturated and resuspended in panning buffer containing insulin, and then sequentially immunopanned at room temperature on three plates containing Ran-2, GalC and O4 antibodies from hybridomal supernatant. The remaining O4^+^GalC^−^ OPCs were removed from the plates with trypsin, resuspended in growth media and seeded at 37 °C on poly-D-Lysine (pDL)-coated flasks to facilitate proliferation. Once sufficient numbers were reached, OPCs were split, plated in differentiation media and allowed to differentiate overnight. Plates were randomly designated for treatment groups and times; treatments were then added to fresh differentiation media and were refreshed as specified per experiment.

### *In vitro* analysis of cell survival

Following 48 h of treatment where treatment was refreshed at 24 h, the medium was removed and differentiating rat OPCs were incubated in PI/FDA (propidium iodide/fluorescein diacetate) for 3 min, rinsed with 1 × PBS and imaged for dead/live cell quantification. TUNEL staining (no. G3250, Promega) was conducted as per the manufacturer’s instructions on cells treated as above.

### Western blot

After 48 h of treatment where treatment was only added at the initiation of the experiment, dOPCs were rinsed twice with sterile 1 × PBS, lysed with ice-cold RIPA buffer containing protease inhibitors (cOmplete mini inhibitor cocktail, Roche) and phosphatase inhibitors (no. P2850 and no. P5726, Sigma), and then scraped and removed to microcentrifuge tubes for incubation on ice for 10 min. Mouse tissue was isolated into microcentrifuge tubes, immediately frozen on dry ice, and then stored at −80 °C until homogenization. The protein concentration of each extract was determined using a BCA protein assay kit (Thermo Scientific Pierce) as per the manufacturer’s instructions. Extracts were denatured in Laemmli buffer with ß-mercaptoethanol, separated by SDS–PAGE and transferred to nitrocellulose. Blots were blocked in 5% non-fat milk in TBST and then incubated in primary antibody in blocking solution. Signal was detected via chemiluminescence (SuperSignal West Dura Extended Duration Substrate, Thermo Scientific Pierce) following horseradish peroxidase-conjugated secondary antibody incubation in blocking buffer. Images have been cropped for presentation. Full-size images are presented in Supplementary Fig. 12. Antibodies to the following were used: 1:500 CHOP (no. MA1-250, Thermo Fisher Pierce), 1:5,000 GAPDH (no. 2118, Cell Signaling), 1:500 p-eIF2α (no. ab32157, Abcam) and 1:1,000 eIF2α (no. 9722 S, Cell Signaling).

### Rat cerebellar sections

Cerebellar explants were prepared from 11-day-old Sprague–Dawley rat pups. Whole brains were extracted and embedded in 1.2% agarose in 1 × PBS before sagittal cerebellar sections were cut at a thickness of 300 μm on a Leica Vibratome. Two to three sections were collected per well, allowed to recover for 2 days, and then grown in HI GM+Hong’s N2+PDGF-AA containing 100 U ml^−1^ IFN-γ (Calbiochem) and 2.5, 5.0 or 10 uM guanabenz (MP Biomedicals) as indicated for 7 days. Half the volume of media, including treatments, were refreshed daily. Slices were stained free-floating for MBP (Covance, SMI99) and then mounted on a slide and coverslipped for immunofluorescence analysis. For electron microscopy and toluidine blue staining, sections were fixed in 2.5% gluteraldehyde, post-fixed in 1% osmium tetroxide, dehydrated and embedded in epoxy resin for examination with a Joel 100CX microscope at 80 kV.

### *GFAP/tTA; TRE/IFN-γ* mice

As previously described^14^, line 110 *GFAP/tTA* mice and line 184 *TRE/IFN-γ* mice, both backcrossed more than 10 times on the C57BL/6 background, were mated to generate double transgenic animals of which both sexes were used. Transcriptional activation of the *TRE/IFN-γ* transgene by tTA was prevented by placing plugged females in cages in which 0.05 mg ml^−1^ doxycycline was added to the drinking water. Pregnant females were removed to normal water at E14.5. Treatment with 4 mg kg^−1^ of guanabenz of every second litter born was initiated on postnatal day 7 in the resulting pups and continued daily until they were being killed at P18; remaining litters were treated similarly with vehicle (0.9% NaCl). All procedures involving animals were performed according to the guidelines of the Institutional Animal Care and Use Committee (IACUC) of the University of Chicago.

### GADD34 mutant mice

GADD34 mutant mice^56^ that were backcrossed more than 10 times on the C57BL/6 background were a gift from Dr David Ron. Mice of both sexes were used.

### Immunofluorescence

Mice were deeply anaesthetized with 2.5% avertin and then transcardially perfused with 0.9% NaCl followed by cold 4% paraformaldehyde. The brain and spinal cord were extracted, post fixed in 4% paraformaldehyde overnight and incubated in 30% sucrose until saturation. Tissue was embedded in optimal temperature cutting compound (OCT) and sectioned at 10 μm. Slides were stored at −80 °C until staining. Before staining, sections were air-dried at room temperature (RT), incubated at −20 °C in acetone and washed twice in 1 × PBS for 5 min each. Sections were then blocked with 5% FBS and 0.1% Triton-X in 1 × PBS for an hour at RT before incubation in primary antibody in blocking solution for 2 h at RT or overnight at 4 °C. Tissue was then incubated in secondary antibody mounting and coverslipping with ProLong Gold antifade reagent with 4,6-diamidino-2-phenylindole (Invitrogen, Carlsbad, CA). Images were visualized with an Olympus IX81 inverted microscope and images taken with a Hamamatsu Orca Flash 4.0 camera. Antibodies to the following were used: 1:1,000 MBP (no. ab7349, Abcam), 1:500 CD3 (no. ab5690, Abcam), 1:100 TPPP (no. PA5-19243, Thermo Fisher/Pierce) and 1:1,000 ASPA (kindly provided by M.A. Namboodiri, Uniformed Services University of the Health Sciences, Bethesda, Maryland).

### Histological stains

Fixed frozen sections were prepared as above and stained with either Mayer’s haematoxylin and eosin or LFB using the standard protocols. Toluidine blue sections were prepared as for electron microscopy as described above and stained using standard protocols.

### Chronic EAE immunization

Preparations of 200-μg MOG_35–55_ peptide emulsified in CFA (no. 263910, BD Biosciences, San Jose, CA) supplemented with 200 μg of *Mycobacterium tuberculosis* (strain H37Ra, BD Biosciences) were injected subcutaneously into the lower flanks of 8-week-old female C57BL/6J mice (Jackson Laboratory, Bar Harbor, MN). Two intraperitoneal (i.p.) injections of 400-ng pertussis toxin each (no. 181, List Biological Laboratories, Denver, CO) were administered 0 and 48 h later. Mice were given i.p. injections of guanabenz or vehicle (sterile 0.9% NaCl) daily beginning PID7. Treatment groups were randomized by assignment before injections on the basis of cage number. Mice were monitored for clinical symptoms beginning PID7 and scored daily (0=healthy, 1=flaccid tail, 2=ataxia and/or paresis of hindlimbs, 3=paralysis of hindlimbs and/or paresis of forelimbs, 4=tetraparalysis, 5=moribund or death). To minimize daily scoring bias, mice were blindly assessed for clinical score before confirmation of treatment dosage and injection. Treatments were administered at the same time every day, ±1–2 h. Data from all mice were included in the study, as per previously established criteria.

### Adoptive transfer EAE immunization

Donor wild-type C57BL/6J females were immunized with MOG_35–55_ and CFA as detailed above to induce an immune response. On PID8, their draining lymph nodes were pulled and isolated cells reactivated in the presence of MOG_35–55_ (20 μg/ml) and IL-12 (10 ng/ml). After 72 h in culture, total cell number and total number of blasts were counted, with blasts typically representing 30% of total cell numbers. The whole cell population was then resuspended in 1xPBS and injected intraperitoneally into 8–10 week old naïve recipient wild-type C57BL/6J females, such that each recipient mouse received 3 × 10^6^ blast cells. Recipient mice were given two IP injections of 200 ng pertussis toxin each 0 and 48 h later and randomly assigned to treatment groups Before injection.

### Relapsing-remitting EAE immunization

Preparations of 50 μg proteolipid protein 139–151 peptide emulsified in CFA supplemented with 200 μg of *Mycobacterium tuberculosis* (as described above) were injected subcutaneously into the lower flanks of 8-week-old female SJL mice (Harlan Laboratories, Indianapolis, IN). Mice were monitored for clinical symptoms beginning PID7 and scored daily as described above. Animals that did not achieve an acute phase of disease (5 out of 50) were removed from the study, as per previously established criteria. Every first remaining mouse was treated intraperitoneally with vehicle (sterile 0.9% NaCl) and every second with 8 mg kg^−1^ of guanabenz daily at the beginning of remission, defined as the second sequential day of reduced clinical score after the peak score of the acute phase. All mice that did not undergo a relapse phase (9 out of 22 vehicle-treated and 9 out of 23 guanabenz-treated) were removed from the study, as per previously established criteria.

### Flow cytometry analysis

Splenic, inguinal lymph nodes and CNS leukocytes isolated from the brain and spinal cords of individual mice perfused with PBS were made into a single-cell suspension as previously described^57^. Flow cytometric analysis and additional analysis was performed by a blinded investigator on cells from individual animals. Cells were stained with two separate panels. For the analysis of immune cells present within the CNS during actively induced EAE, the first analysis panel for T cell populations contained anti-CD45-APC-Cy7 (clone 30-F11), anti-CD3-PerCP (clone 145-2C11), anti-CD4-Pacific Blue (clone RM4-5), anti-CD8alpha-FITC (clone 53-6.7), anti-CD25-APC (clone PC61) and anti-CD44-PE (phycoerythrin; clone IM7). The second analysis panel for antigen-presenting cell populations contained anti-CD45-APC-Cy7 (clone 30-F11), anti-CD3-PerCP (clone 145-2C11), anti-CD11b-Pacific Blue (clone M1/70), anti-CD11c-PE (clone HL3) and anti-CD19-PE-Cy7 (clone ID3). For the analysis of CD4+ T cell populations in the MOG_35–55_ adoptive transfer EAE the first anti-CD45-PE-Cy7 (clone 30-F11), anti-CD3-FITC (clone 145-2C11), anti-CD4-APC/Cy7 (clone RM4-5), anti-CD25-PE (clone PC61), anti-CD44-PE/Cy7 (clone IM7) and AnnexinV-APC. For the second analysis panel total cells were activated in the presence of phorbol myristate acetate (50 ng ml^−1^) and ionomycin (500 ng ml^−1^) for 2 h followed by the addition of brefeldin A (10 μg ml^−1^) for an additional 2 h. The cells were then stained with anti-CD45-PE-Cy7 (clone 30-F11), anti-CD3-FITC (clone 145-2C11), anti-CD4-APC/Cy7 (clone RM4-5), anti-CD25-PE (clone PC61), anti-Ki67-Pacific Blue (clone SolA15), anti-IFN-γ-PerCP/Cy5.5 (clone XMG1.2) and anti-IL-17-APC (clone eBio17B7; eBioscience). Viable cells (1 × 10^6^ cells per tube) were analysed per individual sample using a BD Canto II cytometer (Becton Dickinson), and the data were analysed using BD FACSDiva version 6.1 software (BD Bioscience).

### *Ex vivo* recall and cytokine protein analysis

For *ex vivo* recall cultures, two sets of cultures were set up, with cells isolated as described above from the inguinal lymph nodes and spleens cultured in triplicate wells per individual mouse (*n*=7–10 mice per treatment group) at 5 × 10^5^ cells per well in the presence of anti-CD3 (1 μg ml^−1^), OVA_323–339_ or MOG_35–55_ (20 μg ml^−1^) in the HL-1 medium. For the cellular proliferation cultures, at 24 h post-culture initiation, the wells were pulsed with 1μ Ci of ^3^H-TdR and the cultures were harvested at 72 h and ^3^H-TdR incorporation detected using a Topcount Microplate Scintillation Counter. Results are expressed as the mean counts per minute of triplicate cultures. For cytokine analysis replicate wells were harvested on d+3 of culture or lumbar spinal cord lysates were used and the level of cytokine secreted determined via multiplex Luminex LiquiChip (Millipore).

### Real-time PCR

Total RNA was isolated from snap-frozen lumbar spinal cord using the Aurum Total RNA Mini Kit (Bio-Rad Laboratories). RNA concentration was measured by Nanodrop spectrophotometer and RNA quality was measured using the Agilent 6,000 Nano kit on an Agilent 2,100 bioanalyzer (Agilent Technologies). RNA was reverse-transcribed using the Bio-Rad iScript cDNA Synthesis Kit according to the manufacturer’s instructions, and quantitative real-time PCR was run on a Bio-Rad CFX96 Real-Time PCR detection system as previously described^58^ using the SYBR Green technology. Results were analysed using the Bio-Rad CFX Manager software and presented as the fold induction relative to the reference gene *RPL13A* using the Δ*C*(t) method. Primers used (Integrated DNA Technologies Inc) are as follows: mouse IFN-γ sense primer (5′-GATATCTGGAGGAACTGGCAAAA-3′); mouse IFN-γ antisense primer (5′-CTTCAAAGAGTCTGAGGTAGAAAGAGATAAT-3′); mouse IL-17 sense primer (5′-ATGCTGTTGCTGCTGCTGAG-3′); and mouse IL-17 antisense primer (5′-TTTGGACACGCTGAGCTTTGAG-3′).

### Statistical analysis

Data are presented as mean±s.e.m. unless otherwise noted. Multiple comparisons were statistically evaluated by the mixed results analysis of variance (ANOVA) test or two-way ANOVA test; single comparisons was performed via two-sided unpaired *t*-test. A *P* value <0.05 was considered significant.

## Additional information

**How to cite this article:** Way, S. W. *et al.* Pharmaceutical integrated stress response enhancement protects oligodendrocytes and provides a potential multiple sclerosis therapeutic. *Nat. Commun.* 6:6532 doi: 10.1038/ncomms7532 (2015).

## Supplementary Material

Supplementary Information Supplementary Figures 1-12 and Supplementary Table 1

## References

[b1] Frohman E. M., Racke M. K. & Raine C. S. Multiple sclerosis--the plaque and its pathogenesis. N. Engl. J. Med. 354, 942–955 (2006) . 16510748 10.1056/NEJMra052130

[b2] Dutta R. & Trapp B. D. Mechanisms of neuronal dysfunction and degeneration in multiple sclerosis. Prog. Neurobiol. 93, 1–12 (2011) . 20946934 10.1016/j.pneurobio.2010.09.005 PMC3030928

[b3] Trapp B. D. & Nave K. A. Multiple sclerosis: an immune or neurodegenerative disorder? Annu. Rev. Neurosci. 31, 247–269 (2008) . 18558855 10.1146/annurev.neuro.30.051606.094313

[b4] Hauser S. L., Chan J. R. & Oksenberg J. R. Multiple sclerosis: prospects and promise. Ann. Neurol. 74, 317–327 (2013) . 23955638 10.1002/ana.24009

[b5] Zipp F., Gold R. & Wiendl H. Identification of inflammatory neuronal injury and prevention of neuronal damage in multiple sclerosis: hope for novel therapies? JAMA Neurol. 70, 1569–1574 (2013) . 24145957 10.1001/jamaneurol.2013.4391

[b6] Deshmukh V. A. *et al.* A regenerative approach to the treatment of multiple sclerosis. Nature 502, 327–332 (2013) . 24107995 10.1038/nature12647 PMC4431622

[b7] Bai L. *et al.* Hepatocyte growth factor mediates mesenchymal stem cell-induced recovery in multiple sclerosis models. Nat. Neurosci. 15, 862–870 (2012) . 22610068 10.1038/nn.3109 PMC3427471

[b8] Mei F. *et al.* Micropillar arrays as a high-throughput screening platform for therapeutics in multiple sclerosis. Nat. Med. 20, 954–960 (2014) . 24997607 10.1038/nm.3618 PMC4830134

[b9] Nave K. A. Myelination and the trophic support of long axons. Nat. Rev. Neurosci. 11, 275–283 (2010) . 20216548 10.1038/nrn2797

[b10] Franklin R. J., ffrench-Constant C., Edgar J. M. & Smith K. J. Neuroprotection and repair in multiple sclerosis. Nat. Rev. Neurol. 8, 624–634 (2012) . 23026979 10.1038/nrneurol.2012.200

[b11] Wujek J. R. *et al.* Axon loss in the spinal cord determines permanent neurological disability in an animal model of multiple sclerosis. J. Neuropathol. Exp. Neurol. 61, 23–32 (2002) . 11829341 10.1093/jnen/61.1.23

[b12] Hisahara S. *et al.* Targeted expression of baculovirus p35 caspase inhibitor in oligodendrocytes protects mice against autoimmune-mediated demyelination. EMBO J. 19, 341–348 (2000) . 10654933 10.1093/emboj/19.3.341 PMC305571

[b13] Mc Guire C. *et al.* Oligodendrocyte-specific FADD deletion protects mice from autoimmune-mediated demyelination. J. Immunol. 185, 7646–7653 (2010) . 21068410 10.4049/jimmunol.1000930

[b14] Lin W., Harding H. P., Ron D. & Popko B. Endoplasmic reticulum stress modulates the response of myelinating oligodendrocytes to the immune cytokine interferon-gamma. J. Cell Biol. 169, 603–612 (2005) . 15911877 10.1083/jcb.200502086 PMC2171696

[b15] Donnelly N., Gorman A. M., Gupta S. & Samali A. The eIF2alpha kinases: their structures and functions. Cell Mol. Life Sci. 70, 3493–3511 (2013) . 23354059 10.1007/s00018-012-1252-6 PMC11113696

[b16] Zhang K. & Kaufman R. J. From endoplasmic-reticulum stress to the inflammatory response. Nature 454, 455–462 (2008) . 18650916 10.1038/nature07203 PMC2727659

[b17] Ron D. & Walter P. Signal integration in the endoplasmic reticulum unfolded protein response. Nat. Rev. Mol. Cell Biol. 8, 519–529 (2007) . 17565364 10.1038/nrm2199

[b18] Palam L. R., Baird T. D. & Wek R. C. Phosphorylation of eIF2 facilitates ribosomal bypass of an inhibitory upstream ORF to enhance CHOP translation. J. Biol. Chem. 286, 10939–10949 (2011) . 21285359 10.1074/jbc.M110.216093 PMC3064149

[b19] Cunnea P. *et al.* Expression profiles of endoplasmic reticulum stress-related molecules in demyelinating lesions and multiple sclerosis. Mult. Scler. 17, 808–818 (2011) . 21382862 10.1177/1352458511399114

[b20] Mhaille A. N. *et al.* Increased expression of endoplasmic reticulum stress-related signaling pathway molecules in multiple sclerosis lesions. J. Neuropathol. Exp. Neurol. 67, 200–211 (2008) . 18344911 10.1097/NEN.0b013e318165b239

[b21] Fhlathartaigh M. N. *et al.* Calreticulin and other components of endoplasmic reticulum stress in rat and human inflammatory demyelination. Acta Neuropathol. Commun. 1, 37 (2013) . 24252779 10.1186/2051-5960-1-37 PMC3893522

[b22] Getts M. T., Getts D. R., Kohm A. P. & Miller S. D. Endoplasmic reticulum stress response as a potential therapeutic target in multiple sclerosis. Therapy 5, 631–640 (2008) . 20357912 10.2217/14750708.5.5.631 PMC2847412

[b23] Lin W. & Popko B. Endoplasmic reticulum stress in disorders of myelinating cells. Nat. Neurosci. 12, 379–385 (2009) . 19287390 10.1038/nn.2273 PMC2697061

[b24] Pfeiffer S. E., Warrington A. E. & Bansal R. The oligodendrocyte and its many cellular processes. Trends Cell Biol. 3, 191–197 (1993) . 14731493 10.1016/0962-8924(93)90213-k

[b25] Lin W. *et al.* Oligodendrocyte-specific activation of PERK signaling protects mice against experimental autoimmune encephalomyelitis. J. Neurosci. 33, 5980–5991 (2013) . 23554479 10.1523/JNEUROSCI.1636-12.2013 PMC3654380

[b26] Lin W. *et al.* Enhanced integrated stress response promotes myelinating oligodendrocyte survival in response to interferon-gamma. Am. J. Pathol. 173, 1508–1517 (2008) . 18818381 10.2353/ajpath.2008.080449 PMC2570140

[b27] Baum T. & Shropshire A. T. Studies on the centrally mediated hypotensive activity of guanabenz. Eur. J. Pharmacol. 37, 31–44 (1976) . 6290 10.1016/0014-2999(76)90005-4

[b28] Tsaytler P., Harding H. P., Ron D. & Bertolotti A. Selective inhibition of a regulatory subunit of protein phosphatase 1 restores proteostasis. Science 332, 91–94 (2011) . 21385720 10.1126/science.1201396

[b29] Popko B., Corbin J. G., Baerwald K. D., Dupree J. & Garcia A. M. The effects of interferon-gamma on the central nervous system. Mol. Neurobiol. 14, 19–35 (1997) . 9170099 10.1007/BF02740619 PMC7091409

[b30] Steinman L. Blockade of gamma interferon might be beneficial in MS. Mult. Scler. 7, 275–276 (2001) . 11724441 10.1177/135245850100700501

[b31] Panitch H. S., Hirsch R. L., Haley A. S. & Johnson K. P. Exacerbations of multiple sclerosis in patients treated with gamma interferon. Lancet 1, 893–895 (1987) . 2882294 10.1016/s0140-6736(87)92863-7

[b32] Panitch H. S., Hirsch R. L., Schindler J. & Johnson K. P. Treatment of multiple sclerosis with gamma interferon: exacerbations associated with activation of the immune system. Neurology 37, 1097–1102 (1987) . 3110648 10.1212/wnl.37.7.1097

[b33] Renno T. *et al.* Interferon-gamma in progression to chronic demyelination and neurological deficit following acute EAE. Mol. Cell Neurosci. 12, 376–389 (1998) . 9888990 10.1006/mcne.1998.0725

[b34] Moffett J. R. *et al.* Extensive aspartoacylase expression in the rat central nervous system. Glia 59, 1414–1434 (2011) . 21598311 10.1002/glia.21186 PMC3143213

[b35] Hershfield J. R. *et al.* Aspartoacylase is a regulated nuclear-cytoplasmic enzyme. FASEB J. 20, 2139–2141 (2006) . 16935940 10.1096/fj.05-5358fje

[b36] Kuerten S. & Lehmann P. V. The immune pathogenesis of experimental autoimmune encephalomyelitis: lessons learned for multiple sclerosis? J. Interferon Cytokine Res. 31, 907–916 (2011) . 21936633 10.1089/jir.2011.0072

[b37] Meacham R. H. *et al.* Relationship of guanabenz concentrations in brain and plasma to antihypertensive effect in the spontaneously hypertensive rat. J. Pharmacol. Exp. Ther. 214, 594–598 (1980) . 7400962

[b38] Meacham R. H. *et al.* Pharmacokinetic disposition of guanabenz in the rhesus monkey. Drug Metab. Dispos. 9, 509–514 (1981) . 6120807

[b39] Meacham R. H. *et al.* Disposition of 14C-guanabenz in patients with essential hypertension. Clin. Pharmacol. Ther. 27, 44–52 (1980) . 7351117 10.1038/clpt.1980.7

[b40] Clarner T. *et al.* Myelin debris regulates inflammatory responses in an experimental demyelination animal model and multiple sclerosis lesions. Glia 60, 1468–1480 (2012) . 22689449 10.1002/glia.22367

[b41] Hasnain S. Z., Lourie R., Das I., Chen A. C. & McGuckin M. A. The interplay between endoplasmic reticulum stress and inflammation. Immunol. Cell Biol. 90, 260–270 (2012) . 22249202 10.1038/icb.2011.112 PMC7165805

[b42] Claudio N., Dalet A., Gatti E. & Pierre P. Mapping the crossroads of immune activation and cellular stress response pathways. EMBO J. 32, 1214–1224 (2013) . 23584529 10.1038/emboj.2013.80 PMC3642686

[b43] Kruger K., Lechtermann A., Fobker M., Volker K. & Mooren F. C. Exercise-induced redistribution of T lymphocytes is regulated by adrenergic mechanisms. Brain Behav. Immun. 22, 324–338 (2008) . 17910910 10.1016/j.bbi.2007.08.008

[b44] Bao J. Y., Huang Y., Wang F., Peng Y. P. & Qiu Y. H. Expression of alpha-AR subtypes in T lymphocytes and role of the alpha-ARs in mediating modulation of T cell function. Neuroimmunomodulation 14, 344–353 (2007) . 18463421 10.1159/000129670

[b45] Priyanka H. P. & ThyagaRajan S. Selective modulation of lymphoproliferation and cytokine production via intracellular signaling targets by alpha1- and alpha2-adrenoceptors and estrogen in splenocytes. Int. Immunopharmacol. 17, 774–784 (2013) . 24055020 10.1016/j.intimp.2013.08.020

[b46] Tompkins S. M. *et al.* De novo central nervous system processing of myelin antigen is required for the initiation of experimental autoimmune encephalomyelitis. J. Immunol. 168, 4173–4183 (2002) . 11937578 10.4049/jimmunol.168.8.4173

[b47] Robinson A. P., Rodgers J. M., Goings G. E. & Miller S. D. Characterization of oligodendroglial populations in mouse demyelinating disease using flow cytometry: clues for MS pathogenesis. PLoS ONE 9, e107649 (2014) . 25247590 10.1371/journal.pone.0107649 PMC4172589

[b48] Miller S. D., Karpus W. J. & Davidson T. S. Experimental autoimmune encephalomyelitis in the mouse. Curr. Protoc. Immunol. Chapter 15, (2010) . 10.1002/0471142735.im1501s88 20143314

[b49] Racke M. K. Experimental autoimmune encephalomyelitis (EAE). Curr. Protoc. Neurosci. Chapter 9, (2001) . 10.1002/0471142301.ns0907s14 18428555

[b50] Scheu S. *et al.* Activation of the integrated stress response during T helper cell differentiation. Nat. Immunol. 7, 644–651 (2006) . 16680145 10.1038/ni1338

[b51] Gourdin M. *et al.* The effect of clonidine, an alpha-2 adrenergic receptor agonist, on inflammatory response and postischemic endothelium function during early reperfusion in healthy volunteers. J. Cardiovasc. Pharmacol. 60, 553–560 (2012) . 22987052 10.1097/FJC.0b013e31827303fa

[b52] Romero-Sandoval E. A., McCall C. & Eisenach J. C. Alpha2-adrenoceptor stimulation transforms immune responses in neuritis and blocks neuritis-induced pain. J. Neurosci. 25, 8988–8994 (2005) . 16192389 10.1523/JNEUROSCI.2995-05.2005 PMC6725591

[b53] Boyce M. *et al.* A selective inhibitor of eIF2alpha dephosphorylation protects cells from ER stress. Science 307, 935–939 (2005) . 15705855 10.1126/science.1101902

[b54] Novoa I. *et al.* Stress-induced gene expression requires programmed recovery from translational repression. EMBO J. 22, 1180–1187 (2003) . 12606582 10.1093/emboj/cdg112 PMC150345

[b55] Dugas J. C., Tai Y. C., Speed T. P., Ngai J. & Barres B. A. Functional genomic analysis of oligodendrocyte differentiation. J. Neurosci. 26, 10967–10983 (2006) . 17065439 10.1523/JNEUROSCI.2572-06.2006 PMC6674672

[b56] Marciniak S. J. *et al.* CHOP induces death by promoting protein synthesis and oxidation in the stressed endoplasmic reticulum. Genes Dev. 18, 3066–3077 (2004) . 15601821 10.1101/gad.1250704 PMC535917

[b57] McMahon E. J., Bailey S. L., Castenada C. V., Waldner H. & Miller S. D. Epitope spreading initiates in the CNS in two mouse models of multiple sclerosis. Nat. Med. 11, 335–339 (2005) . 15735651 10.1038/nm1202

[b58] Lin W. *et al.* Interferon-gamma inhibits central nervous system remyelination through a process modulated by endoplasmic reticulum stress. Brain 129, 1306–1318 (2006) . 16504972 10.1093/brain/awl044

