# Supplementary material for: Pharmaceutical integrated stress response enhancement protects oligodendrocytes and provides a potential multiple sclerosis therapeutic
Source: Nat Commun. 2015 Mar 13;6:6532. doi: 10.1038/ncomms7532 (PMC4360920; doi:10.1038/ncomms7532)
Supplement: Supplementary Information — Supplementary Figures 1-12 and Supplementary Table 1 [file ncomms7532-s1.pdf]

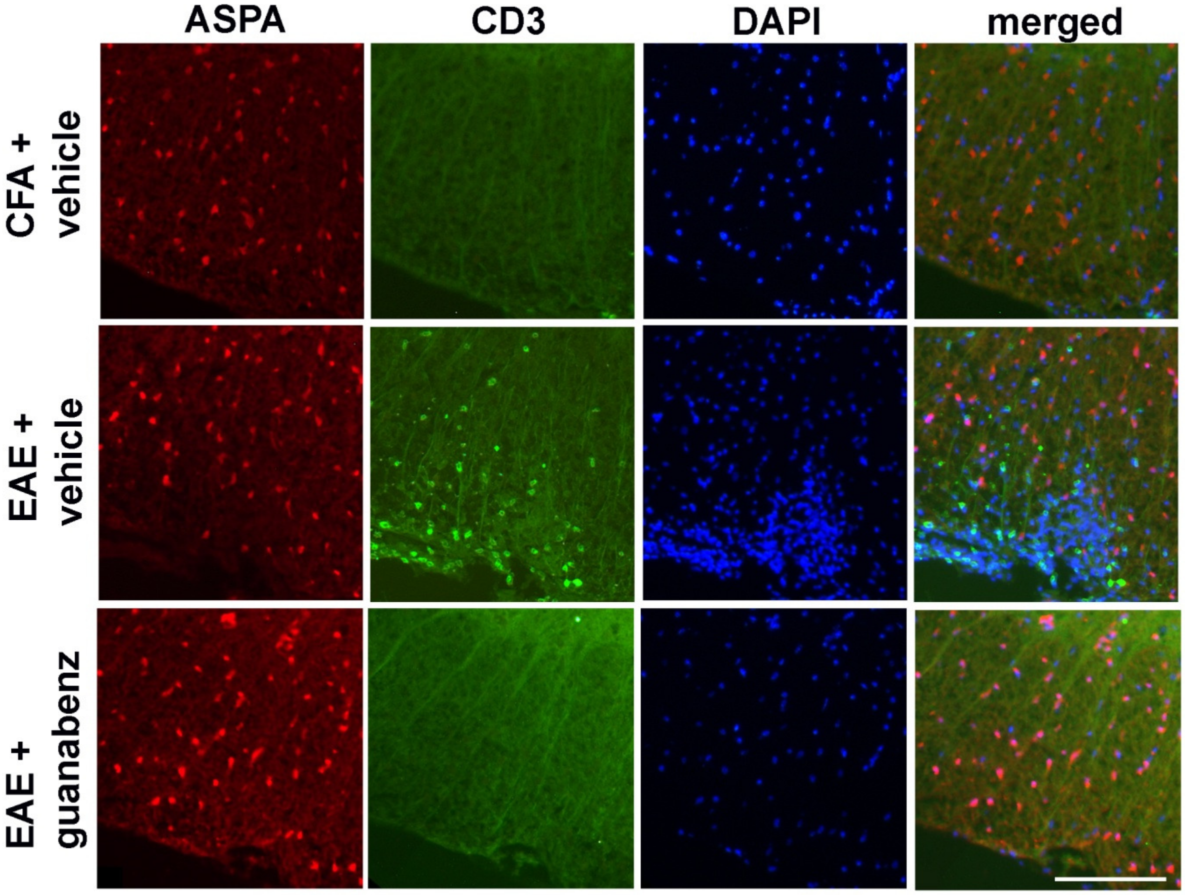

Supplementary Figure 1. Guanabenz treatment protects mature oligodendrocytes and alters T cell presence in EAE mice. Immunostaining of lumbar spinal cord sections of PID15 chronic EAE mice with CD3, a T cell marker, and ASPA, a mature oligodendrocyte marker. Images represent 5 or 6 mice per group. Scale bar, 100 µm.

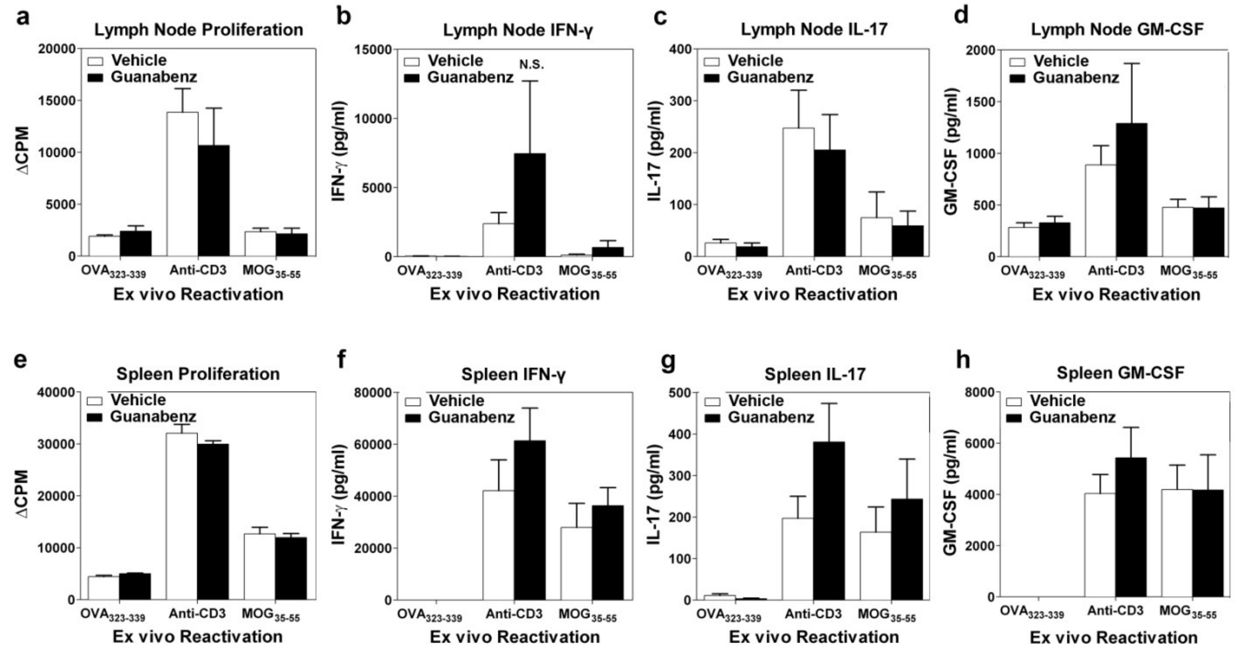

Supplementary Figure 2. Guanabenz treatment does not affect immune cell proliferation or cytokine production in the lymph nodes or spleen of mice with chronic EAE. The level of cellular proliferation (via tritiated thymidine incorporation) and the level of IFN-γ, IL-17, and GM-CSF production was assessed from inguinal lymph node cells **(a-d)** and splenocytes **(e-h)** isolated from PID15 chronic EAE mice after culture with anti-CD3 (1 µg/ml), $OVA_{323-339}$, or MOG_{35-55} (20 µg/ml) for 72 hours. Data in (a-h) are representative of 4 mice per group; experiment performed twice.

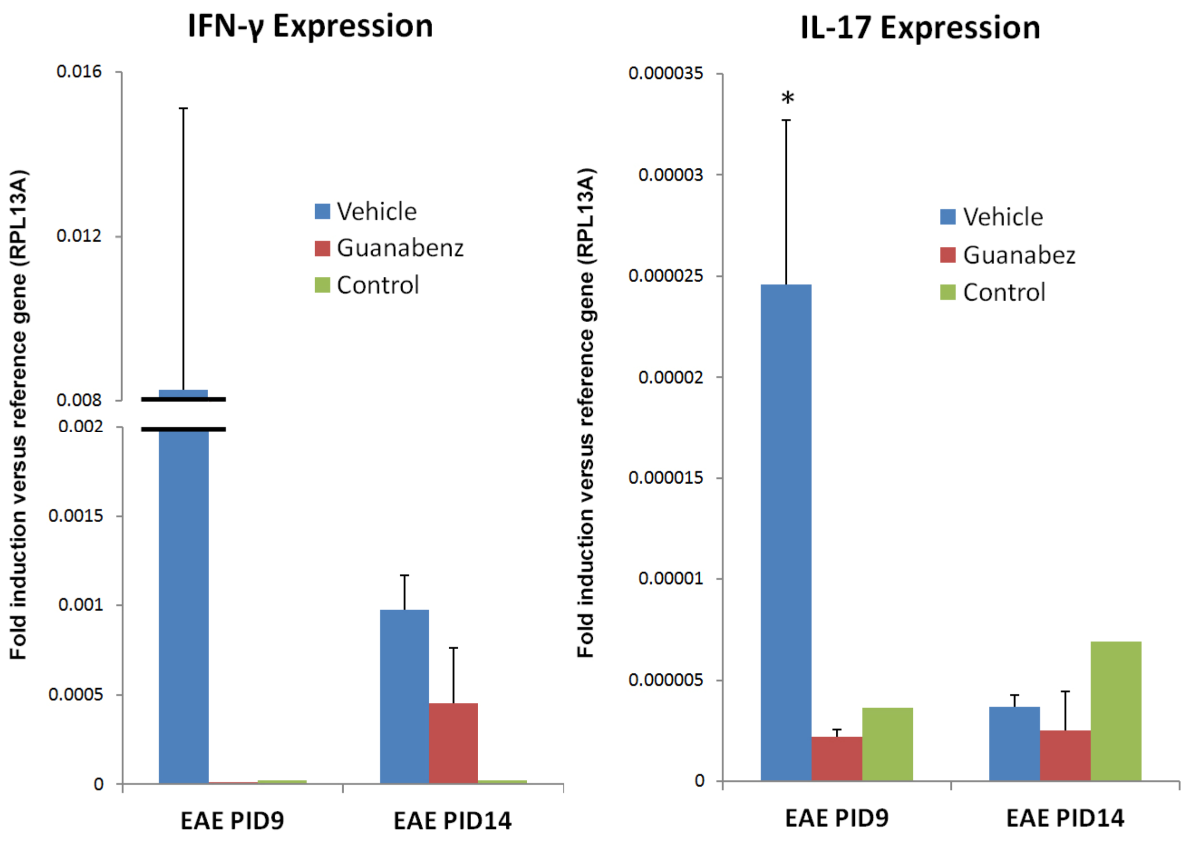

Supplementary Figure 3. Real-time PCR analysis of the pro-inflammatory cytokines IFN-γ and IL-17 in lumbar spinal cord samples. Total RNA was analyzed from the lumbar spinal cords of EAE mice treated with vehicle or guanabenz and WT control mice. Results are presented as the fold induction relative to the reference gene RPL13A using the ΔC(t) method. n=3 per treated group. *$p<0.05$, data presented as mean ± s.e.m.

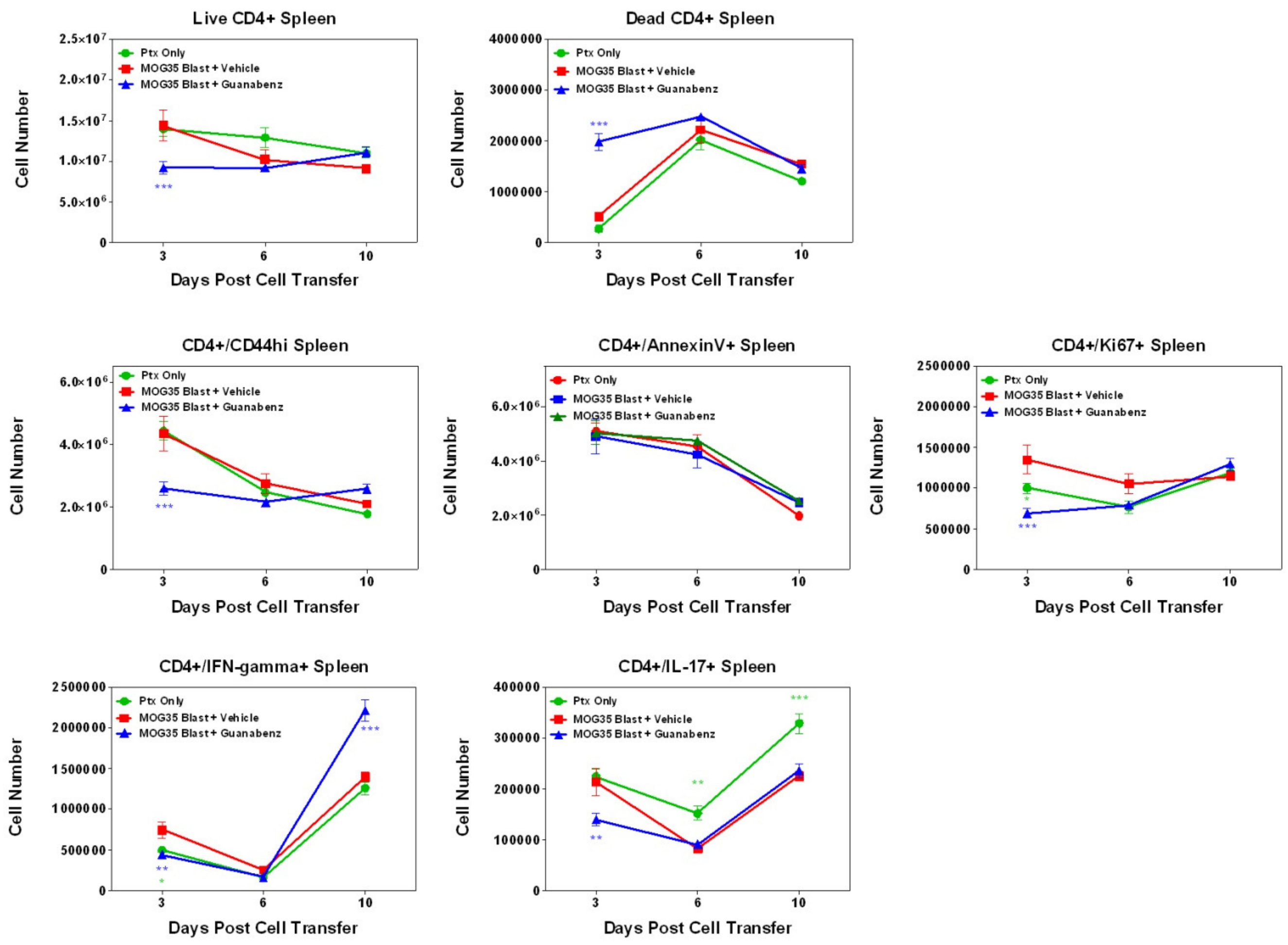

Supplementary Figure 4. Spleen data from adoptive transfer EAE. On days 3, 6, and 10 the spleen was collected from four representative mice for each treatment group and the number of total live CD4+ T cells, dead CD4+ T cells, AnnexinV+ CD4+ T cells, Ki67+ CD4+ T cells, CD44hi CD4+ T cells, IFN-γ+ CD4+ T cells, and IL-17+ CD4+ T cells was assessed via flow cytometry. The data is present as the average number of cells over time. Data are representative of n=4 mice per group and two-way ANOVA analysis was completed. *$p<0.05$, **$p<0.01$, ***$p<0.001$, as compared with vehicle-treated mice. Data represents average of 4 mice per group, presented as mean ± s.e.m.

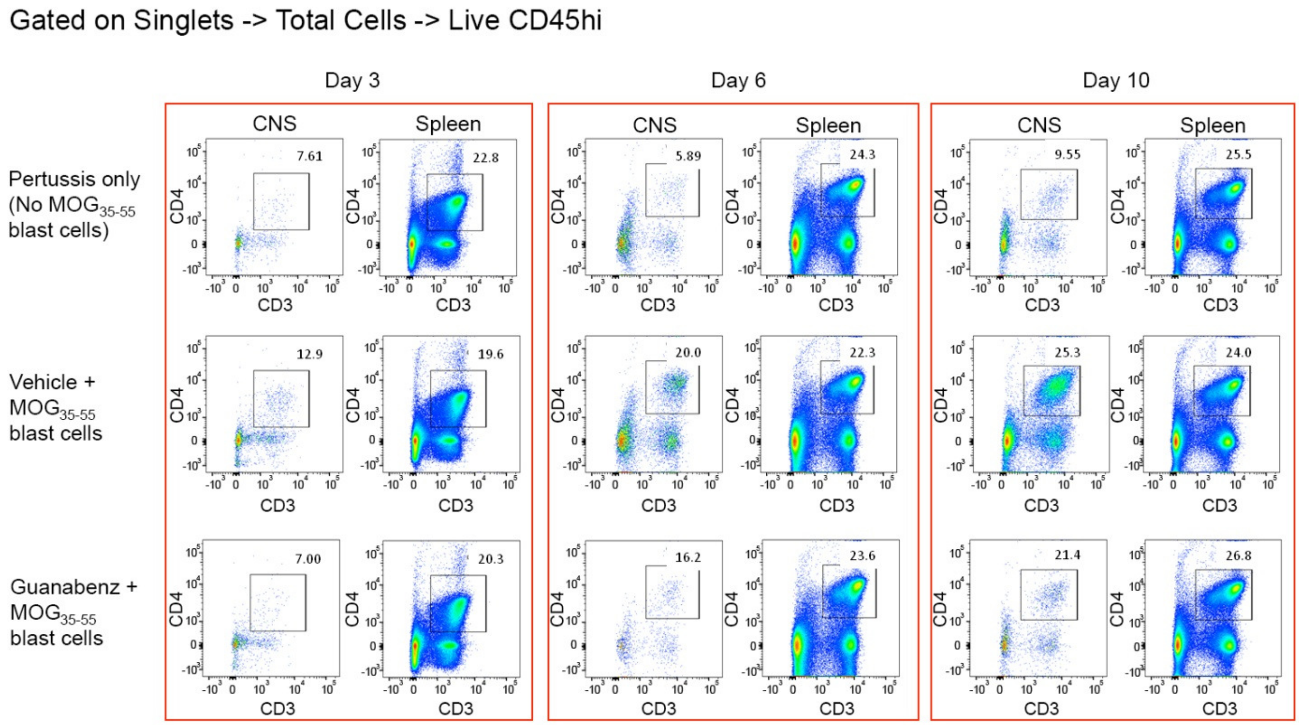

Supplementary Figure 5. Flow analysis for the percentage of CD4+ T cells following guanabenz treatment during adoptive transfer EAE. The data presents the representative flow cytometric plots determining the percentage of CD4+ T cells present within the spleen and CNS as denoted. The calculated number of CD4+ T cells is presented in Figure 6g for the CNS and Supplementary Fig. 4 for the spleen. The gating scheme used is listed on the figure.

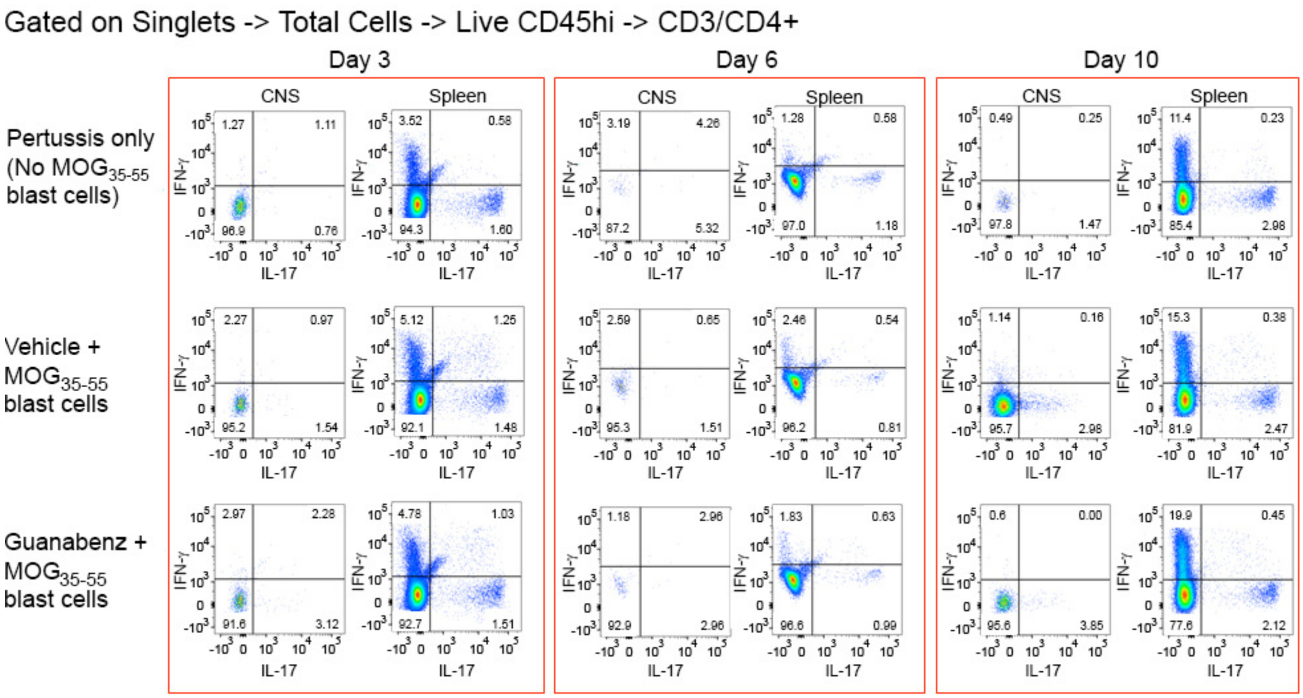

Supplementary Figure 6. Flow analysis for the percentage of IFN-γ+/ and IL-17+/CD4+ T cells following guanabenz treatment during adoptive transfer EAE. The data presents the representative flow cytometric plots determining the percentage of IFN- γ+/CD4+ and IL-17+/CD4+T cells present within the spleen and CNS as denoted. The calculated number of IFN-γ+/CD4+ and IL-17+/CD4+ T cells is presented in Figure 6l and m respectively for the CNS and Supplementary Fig. 4 for the spleen. The gating scheme used is listed on the figure.

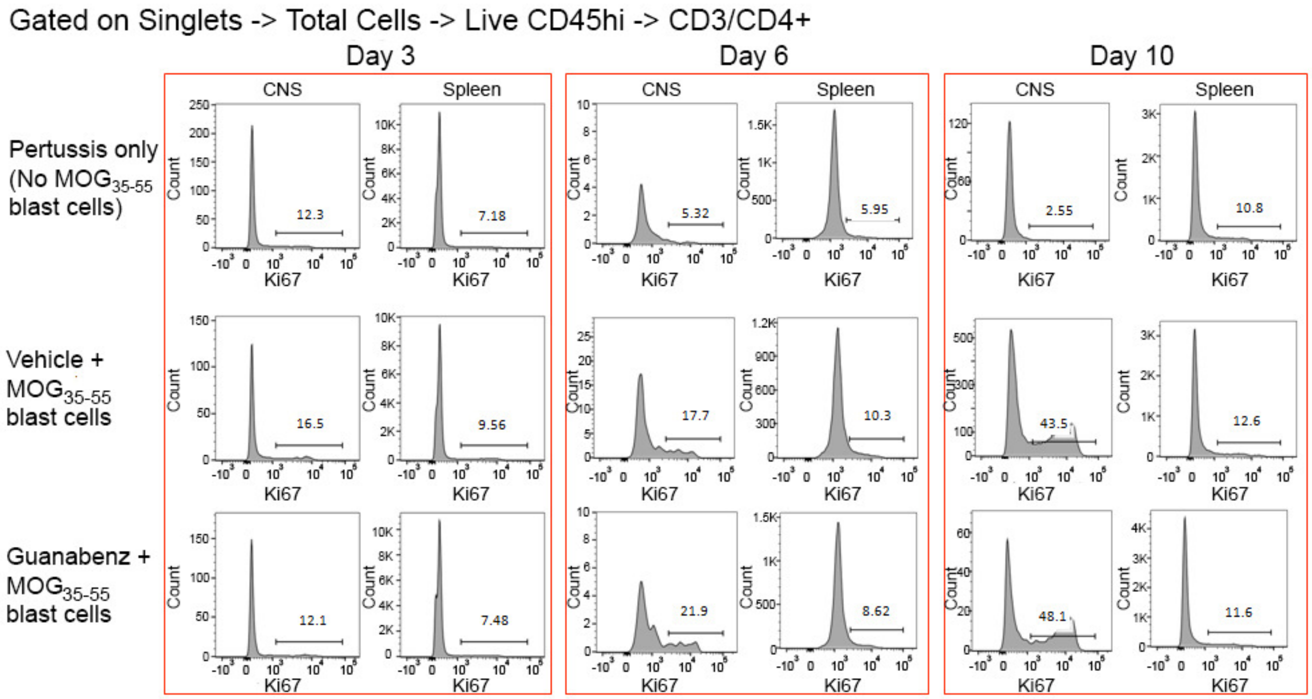

Supplementary Figure 7. Flow analysis for the percentage of Ki67+/CD4+ T cells following guanabenz treatment during adoptive transfer EAE. The data presents the representative flow cytometric plots determining the percentage of Ki67+/CD4+ T cells present within the spleen and CNS as denoted. The calculated number of Ki67+/CD4+ T cells is presented in Figure 6j for the CNS and Supplementary Fig. 4 for the spleen. The gating scheme used is listed on the figure.

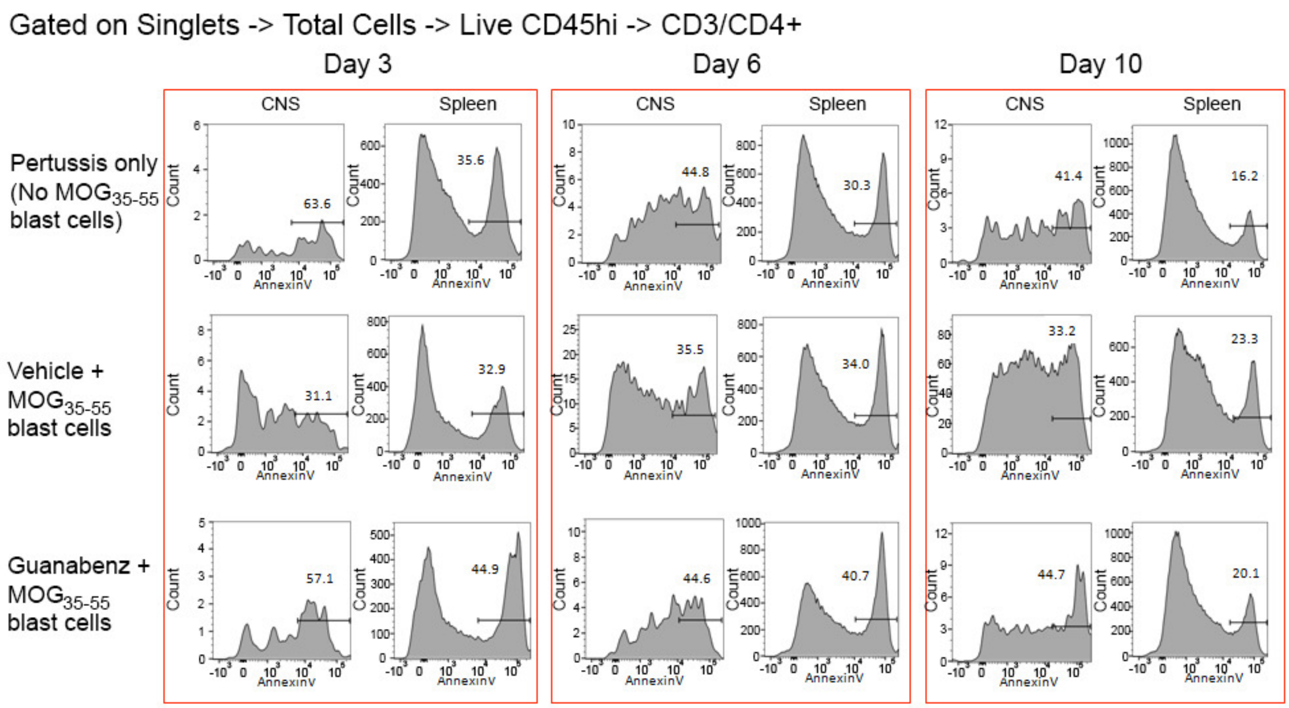

Supplementary Figure 8. Flow analysis for the percentage of AnnexinV+/CD4+ T cells following guanabenz treatment during adoptive transfer EAE. The data presents the representative flow cytometric plots determining the percentage of AnnexinV+/CD4+ T cells present within the spleen and CNS as denoted. The calculated number of AnnexinV+/CD4+ T cells is presented in Figure 6i for the CNS and Supplementary Fig. 4 for the spleen. The gating scheme used is listed on the figure.

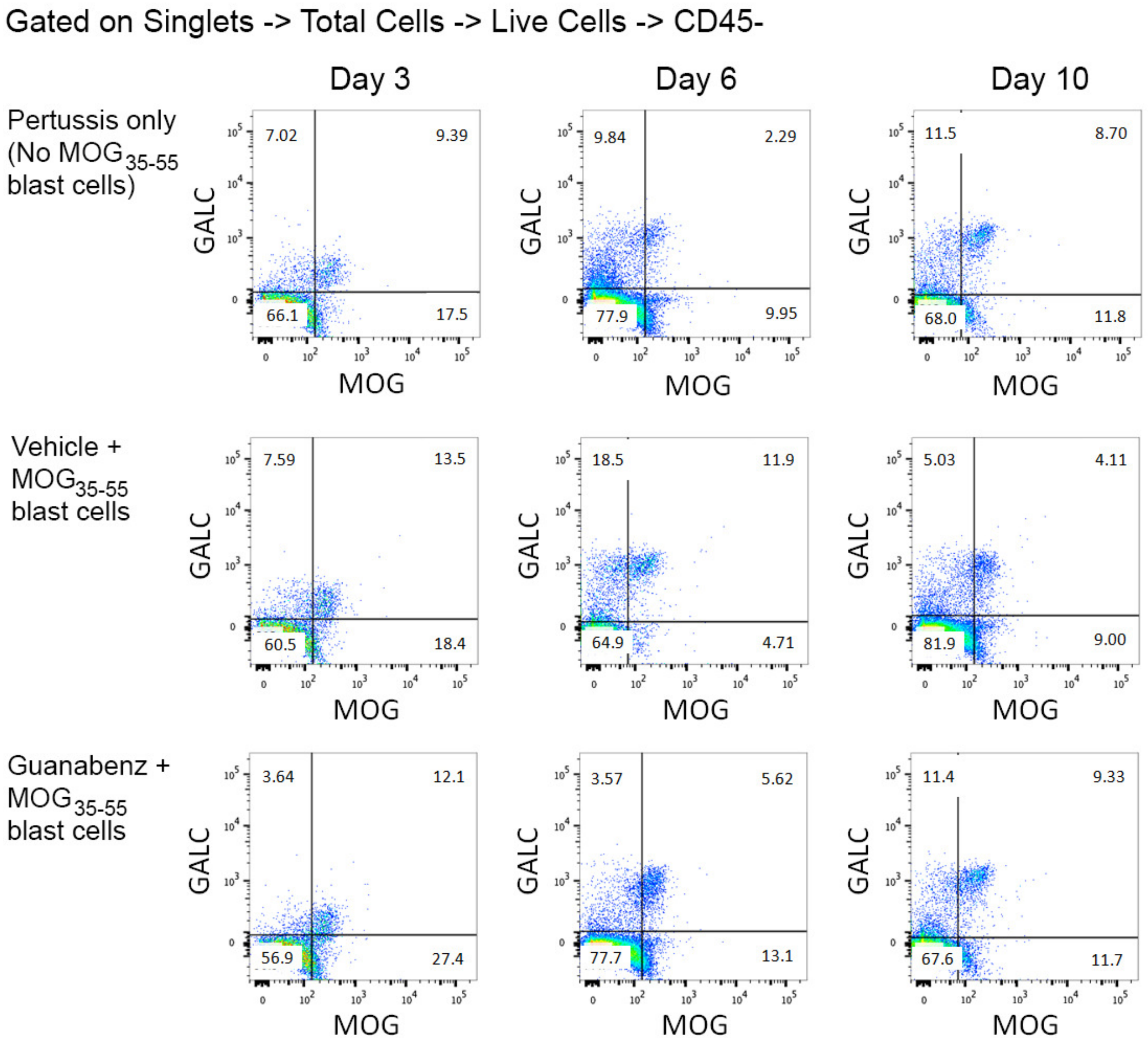

Supplementary Figure 9. Flow analysis for the percentage of GALC+/MOG+ mature oligodendrocytes following guanabenz treatment during adoptive transfer EAE. The data presents the representative flow cytometric plots determining the percentage of GALC+/MOG+ mature oligodendrocytes present within the CNS as denoted. The calculated number of GALC+/MOG+ live mature oligodendrocytes in the CNS is presented in Figure 6n. The gating scheme used is listed on the figure.

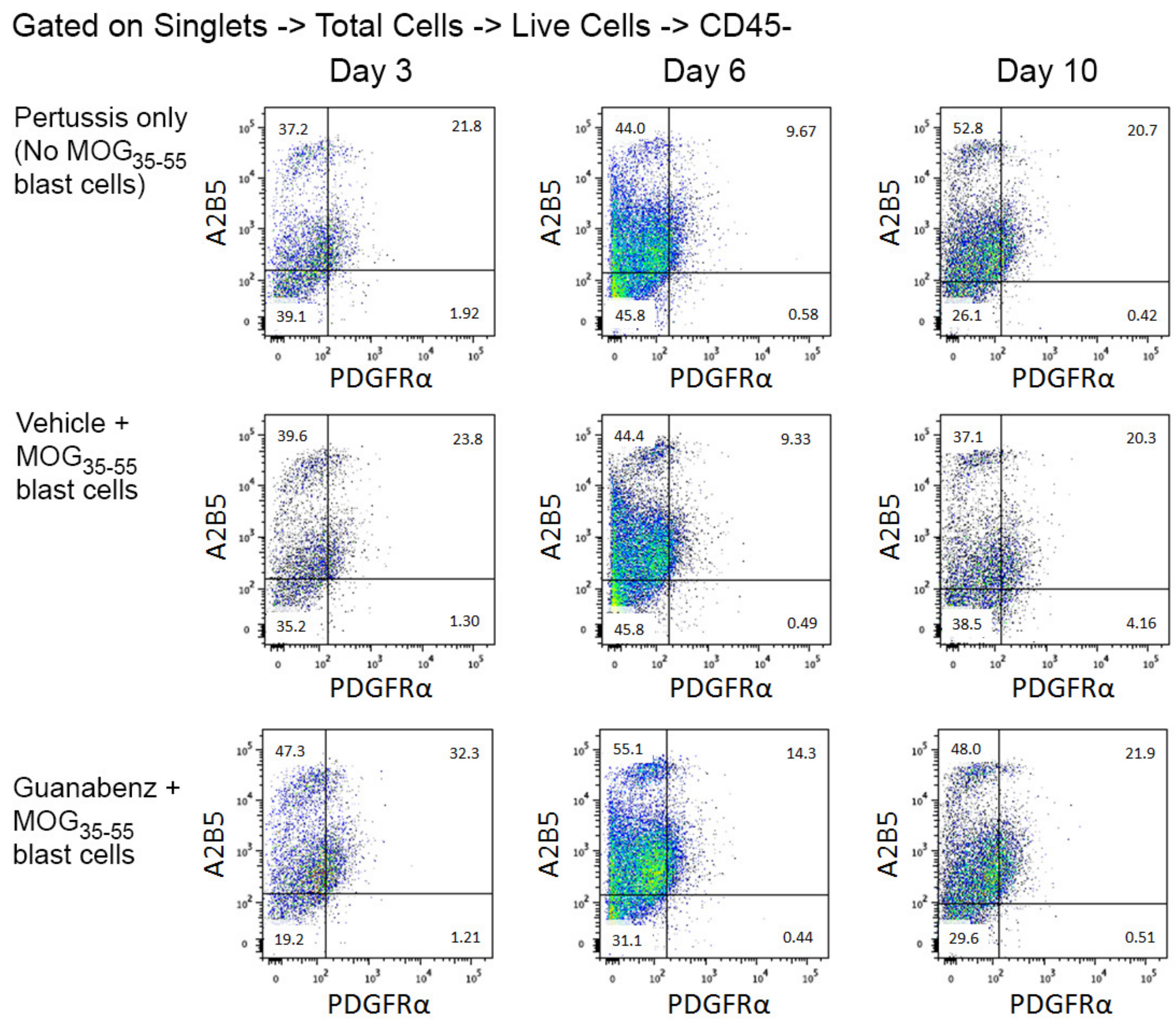

Supplementary Figure 10. Flow analysis for the percentage of A2B5+/PDGFRα+ early OPCs following guanabenz treatment during adoptive transfer EAE.

The data presents the representative flow cytometric plots determining the percentage of A2B5+/PDGFRα+ early OPCs present within the CNS as denoted. The calculated number of A2B5+/PDGFRα+ live early OPCs in the CNS is presented in Figure 6o. The gating scheme used is listed on the figure.

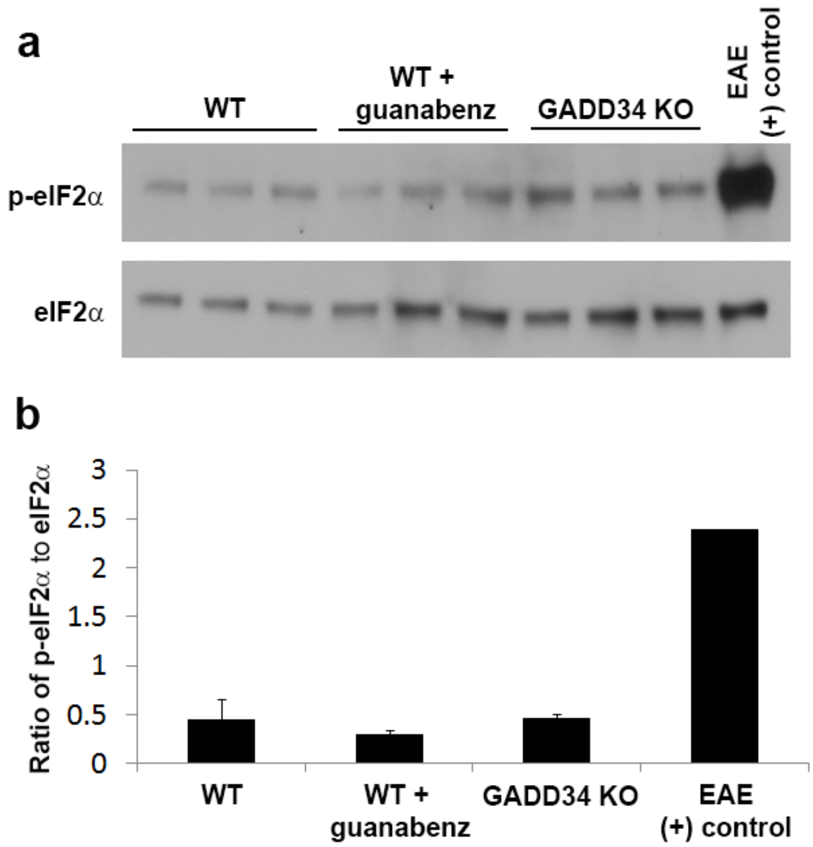

Supplementary Figure 11. Unstressed GADD34 KO mice and guanabenz-treated WT mice display normal levels of p-eIF2α. Lysates of three groups of mice were analyzed for p-eIF2α expression as a ratio of total eIF2α expression: three-month old wildtype C57BL/6J mice (WT), WT mice treated with a single dose of 8 mg/kg guanabenz and sacrificed 24 hours later (WT + guanabenz), and three-month old GADD34 KO mice (GADD34 KO). Mice immunized with MOG_{35-55} peptide/CFA to induce EAE were taken at peak of disease as a positive control of p-eIF2α expression (EAE (+) control). (a) Immunoblot of n=3 per group plus positive control. (b) Quantification of immunoblot shown in (a), represented as ratio of p-eIF2α over total eIF2α expression. Data presented as average ± s.e.m.

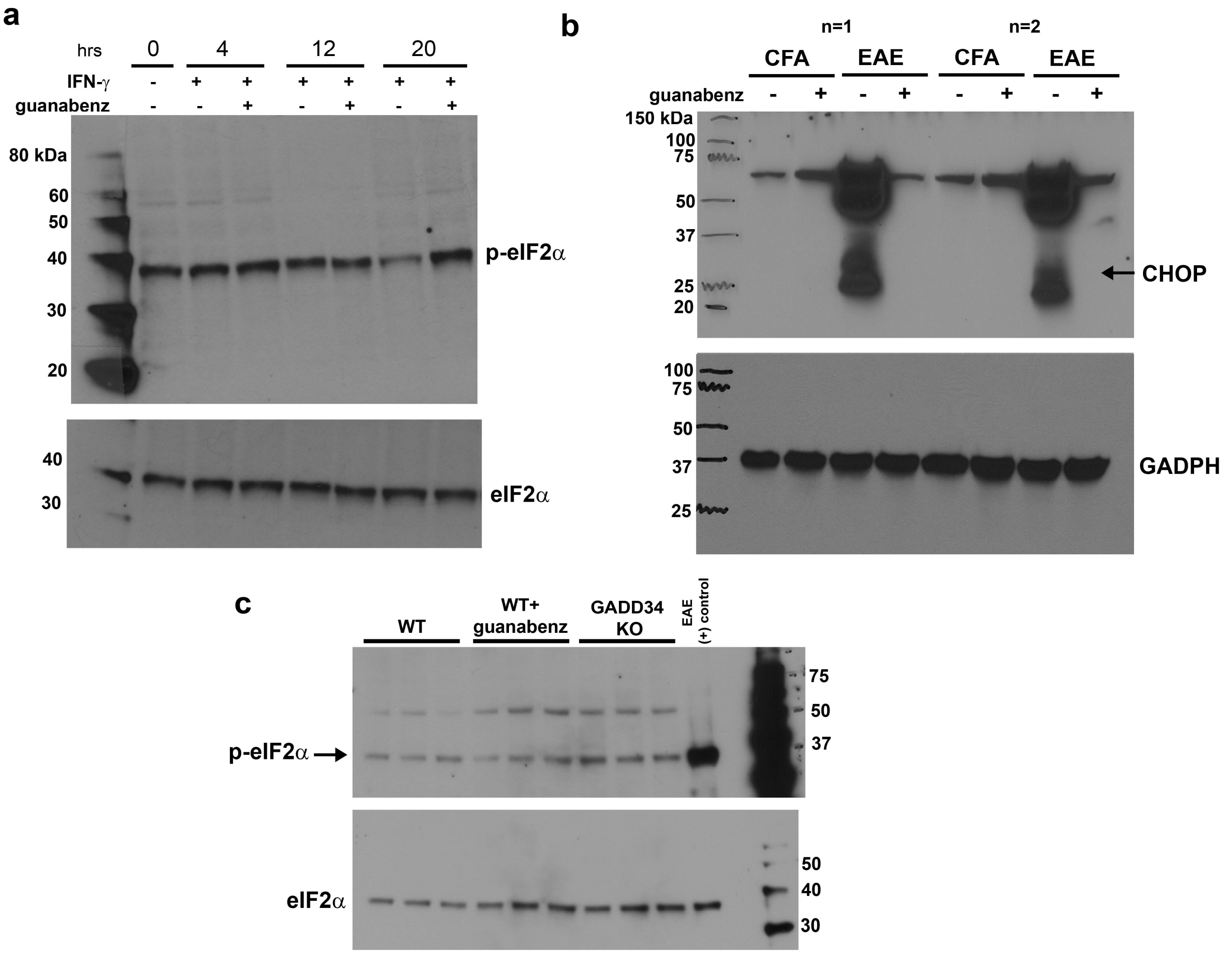

Supplementary Figure 12. Full scans of western blots. Full scans corresponding to **(a)** Figure 1c, showing p-eIF2α and eIF2α, **(b)** Figure 5h, showing CHOP and GADPH, two out of four n tested, and **(c)** Supplementary Fig. 11, showing p-eIF2α and eIF2α.

Dosage	Sample	2 hours	4 hours	12 hours
4 mg/kg	Brain	2,113 ± 1,932 nM (488 ± 446 ng/ml)	1,102 ± 522 nM 255 ± 121 ng/ml)	56 ± 76 nM (13 ± 17 ng/ml)
	Serum	198 ± 235 nM (46 ± 54 ng/ml)	47 ± 27 nM (11 ± 6 ng/ml)	BQL
8 mg/kg	Brain	4,461 ± 2,043 nM (1,031 ± 472 ng/ml)	3,289 ± 2,693 nM (760 ± 622 ng/ml)	21 ± 32 nM (5 ± 8 ng/ml)
	Serum	228 ± 262 nM (53 + 60 ng/ml)	161 ± 213 nM (37 + 49 ng/ml)	BQL
16 mg/kg	Brain	21,700 ± 11,319 nM (5,013 ± 2,615 ng/ml)	5,895 ± 1,421 nM (1,362 ± 328 ng/ml)	95 ± 18 nM (22 ± 4 ng/ml)
	Serum	1,293 ± 1,172 nM (299 ± 271 ng/ml)	192 ± 48 nM (44 ± 11 ng/ml)	BQL

Supplementary Table 1. Pharmacokinetic analysis of the brain and serum of mice with chronic EAE treated with guanabenz. Samples from chronic EAE mice were collected at PID 42 following the termination of the study shown in Figure 4, at the time indicated after the administration of the final dose. A bioanalysis to determine drug exposure was performed by Cerep using LC/MS/MS. BQL: below quantifiable level, for these experiments the limit of quantitation was 1 ng/ml. The serum exposures were evaluated from two separate experiments; brain data were obtained from a single experiment. Data represents average of 3-4 mice per time point and are shown from the second experiment in which both serum and brain samples were collected. Serum data from the second experiment was similar to that observed in the first experiment.
